# Behavioural effects of oral cannabidiol (CBD) treatment in the *superoxide dismutase 1 G93 A* (*SOD1*^*G93 A*^) mouse model of amyotrophic lateral sclerosis

**DOI:** 10.1007/s00213-025-06785-z

**Published:** 2025-04-14

**Authors:** Sandip Ghimire, Fabian Kreilaus, Rossana Rosa Porto, Lyndsey L. Anderson, Justin J. Yerbury, Jonathon C. Arnold, Tim Karl

**Affiliations:** 1 School of Medicine, Western Sydney University, Campbelltown, NSW 2560 Australia; 2 Lambert Initiative for Cannabinoid Therapeutics, Brain and Mind Centre, The University of Sydney, Sydney, NSW Australia; 3 Discipline of Pharmacology, Sydney Pharmacy School, Faculty of Medicine and Health, The University of Sydney, Sydney, NSW Australia; 4 Illawarra Health and Medical Research Institute, Wollongong, NSW Australia; 5 Molecular Horizons and School of Chemistry and Molecular Bioscience, University of Wollongong, Wollongong, NSW Australia

**Keywords:** Amyotrophic lateral sclerosis, Cannabidiol, Oral treatment, *SOD1*^*G93A*^ transgenic mouse model, Behaviour, Cannabinoid therapy

## Abstract

**Background:**

Amyotrophic lateral sclerosis (ALS) is a progressive neurodegenerative disease affecting voluntary muscle movement as well as cognitive and other behavioural domains at later disease stages. No effective treatment for ALS is currently available. Elevated neuroinflammation, oxidative stress and alterations to the endocannabinoid system are evident in ALS. The phytocannabinoid cannabidiol (CBD) has anti-inflammatory and anti-oxidant properties. Thus, we evaluated the remedial effects of chronic oral cannabidiol (CBD) treatment on ALS-relevant behavioural domains in the *copper-zinc superoxide dismutase 1* (*SOD1*) mouse model of ALS that carries a *G93A* mutation (*SOD1*^*G93A*^).

**Methods:**

Male and female *SOD1*^*G93A*^ and wild type-like (WT) littermates were fed either a control (CHOW) or CBD-enriched chow diet (equivalent to a dose of 36 mg/kg per day) beginning from 10 weeks of age. Bodyweight and motor performance were recorded weekly from 11 to 19 weeks and open field behaviours at 12 and 18 weeks. Mice were also tested for prepulse inhibition (PPI), social behaviours, as well as fear-associated memory.

**Results:**

CBD treatment ameliorated the bodyweight loss in female *SOD1*^*G93A*^ mice, tended to reinstate sociability in *SOD1*^*G93A*^ males, strengthened social recognition memory in *SOD1*^*G93A*^ females, and improved the PPI response in younger *SOD1*^*G93A*^ females at higher prepulse intensities. CBD had no effect on motor impairments but instead reversed the anxiolytic-like phenotype of 12-week-old male *SOD1*^*G93A*^ mice and decreased the acoustic startle response and strengthened cue *freezing* in male mice.

**Conclusion:**

Thus, the current remedial oral dose of CBD delayed disease progression (inferred by bodyweight) in both male and female mice and improve specific cognitive deficits of *SOD1*^*G93A*^ mice in a sex specific manner without altering the motor phenotype.

**Supplementary Information:**

The online version contains supplementary material available at 10.1007/s00213-025-06785-z.

## Introduction

Amyotrophic lateral sclerosis (ALS) or motor neuron disease is a characterised by the loss of both upper and lower motor neurons, advancing to a point where the voluntary movement of skeletal muscle is severely impacted. Pathological mechanisms of ALS are believed to involve neuroinflammation (Schiffer et al. 1996), oxidative stress (Barber and Shaw 2010; Ferrante et al. 1997), protein misfolding (Piao et al. 2003; Wang et al. 2008) and protein homeostasis imbalance (Ciryam et al. 2017; Yerbury et al. 2019). Consistent with many of these changes also occurring outside the motor cortex and spinal cord, ALS patients exhibit cognitive and behavioural changes during disease progression including anxiety and executive function deficits (Abrahams et al. 2005). Overlap in pathogenic genes [e.g. *chromosome 9 open reading frame 72*—*C9ORF72*; (Renton et al. 2011)] associated with both ALS and frontotemporal dementia (FTD) supports the idea of the ALS-frontotemporal spectrum disorder that encompasses symptoms ranging from primarily motor deficits through to primarily cognitive deficits (Strong et al. 2017).

Genetic mutations are observed in 5–10% of ALS cases, with approximately 20% of these caused by mutations in *copper-zinc superoxide dismutase 1* (*SOD1*). SOD1 is a ubiquitous cytoplasmic enzyme that catalyzes the breakdown of reactive oxygen species (ROS) preventing harmful oxidative stress, which is of particular importance to neurons that have high energy demands (Rosen et al. 1993). Mutation in the *SOD1* gene have been modelled in mice with the most widely studied being the transgenic *SOD1*^*G93 A*^ mouse that overexpresses human mutant *SOD1* (change from glycine to alanine at residue 93). This mouse is characterised by weight loss, rapid and robust motor degeneration, and also displays several brain pathologies evident in human ALS (gliosis and loss of motor neurons (Gurney et al. 1994)). Furthermore, sex-specific changes in sociability and sensorimotor gating as well as anxiety-like behaviours have been found in the *SOD1*^*G93 A*^ mouse model (Guerra et al. 2021; Kreilaus et al. 2020).

The modest effect of FDA-approved therapeutics for ALS [riluzole, edaravone and nuedexta; e.g. Lacomblez et al. 1996; Smith et al. 2017)] and the failure of the majority of repurposed pharmaceutical [e.g. dexpramipexole, coenzyme Q10, erythropoietin, ceftriaxone; see e.g. Berry et al. 2013; Cudkowicz et al. 1997; Ferrante et al. 2005)] highlight the need to identify and evaluate novel compounds to treat ALS. Cannabidiol (CBD) is a plant-derived cannabinoid known to have properties that are anti-oxidant (Hampson et al. 1998), anti-inflammatory (Esposito et al. 2007; Mori et al. 2017) and neuroprotective by stimulating pro-survival factors such as brain-derived neurotrophic factor (BDNF) (Esposito et al. 2011; Mori et al. 2017). CBD also interacts with the endocannabinoid system, with emerging evidence suggesting that this system is altered in human ALS (i.e. increased spinal cord cannabinoid receptor 2 density) as well as the *SOD1*^*G93 A*^ mouse model (abnormal striatal cannabinoid receptor 1 sensitivity) (Rossi et al. 2010; Yiangou et al. 2006). Purified CBD has shown efficacy to improve cognitive deficits in other mouse models of neurodegenerative diseases such as Alzheimer’s disease (Cheng et al. 2014a, b; Coles et al. 2020; Watt et al. 2020) but its therapeutic potential for the treatment of ALS has not been considered yet. A combination treatment strategy with CBD and the main psychoactive component of cannabis, i.e. Δ^9^-tetrahydrocannabinol (THC), has previously been tested in *SOD1*^*G93 A*^ transgenic mice and resulted in a mild attenuation of the progression of neurological deficits and improved animal survival but behaviour was not characterised in any detail (Moreno‐Martet et al. 2014).

Thus, the aim of the current study was the evaluation of the therapeutic effectiveness of purified CBD to treat ALS-relevant behavioural symptoms in *SOD1*^*G93 A*^ transgenic mice using oral administration. To investigate potential sex-dependent effects, male and female *SOD1*^*G93 A*^ transgenic mice had access to either normal chow diet or CBD-enriched chow diet (equivalent to a dose of 36 mg/kg per day). Treatment started from 10 weeks of age, prior to the onset of severe motor impairments. The study design allowed the evaluation of disease-relevant symptoms beyond the established motor phenotype and included the testing of anxiety, cognition, sensorimotor gating, and social behaviours.

## Materials and methods

### Animals

Male and female *superoxide dismutase 1 G93 A* transgenic [*SOD1*^*G93 A*^: B6-Tg(SOD1-G93 A)1GUr/j] and wild type-like (WT) littermates were used for this study. Animal numbers were as follows: WT Control male *n* = 9; WT Control female *n* = 10; *SOD1*^*G93 A*^ Control male *n* = 8; *SOD1*^*G93 A*^ Control female *n* = 8; WT CBD male *n* = 7; WT CBD female *n* = 13; *SOD1*^*G93 A*^ CBD male *n* = 11; *SOD1*^*G93 A*^ CBD female *n* = 9. Animals were bred at the Australian BioResources (ABR Moss Vale, Australia) where they were housed in individually ventilated (IVC) cages (Type Mouse Version 1; Airlaw, Smithfield, Australia; air change: 90–120 times per hour averaged; passive exhaust ventilation system). Post weaning (PND21), test animals were shipped to the animal facility of the School of Medicine, Western Sydney University (Australia). Animals were housed in groups of 2–3 in IVC cages (GM500 Green, Techniplast Australia Pty Ltd, Rydalmere, Australia) under a 12:12 h light:dark cycle (white light illumination from 0900 and red light illumination from 2100) using corncob bedding (PuraCob Premium: Able Scientific, Perth, Australia). Tissue paper and cardboard (Crink-l’Nest, Kraft) were provided for nesting. Prior to treatment start, all mice were provided with water and standard lab chow (Rat and Mouse Maintenance Pellets; Gordon's Speciality Stockfeeds, Yanderra, Australia) ad libitum. Cages were changed fortnightly. For the social preference test, sex-matched, adult A/JArc mice from the Animal Resources Centre (ARC: Cunning Vale, Australia) were used as social conspecifics.

All research and animal care procedures were approved by the Western Sydney University Animal Care and Ethics Committee (#12905) and were in accordance with the Australian Code of Practice for the Care and Use of Animals for Scientific Purposes.

### Cannabidiol chow treatment

Beginning at 10 weeks of age, mice had only access to either a normal chow control diet (control) or a CBD-enriched chow diet (CBD) as described previously (Anderson et al. 2019, 2020). This ‘oral treatment’ continued until the end of all behavioural testing. Pellets were prepared using chow powder (provided by Gordon's Speciality Stockfeeds, powder identical to the Rat and Mouse Maintenance Pellets) and in case of CBD treatment, 200 mg of purified CBD powder (THC Pharm GmbH, Frankfurt am Main, Germany) was added per 1 kg of dry chow powder. This was then mixed thoroughly for 10 min after which sterile distilled water was added (approximately 750 ml water for 1 kg of chow powder). Control and CBD pellets were manually formed into cylinders approximately 1 cm in diameter and 3–4 cm in length and left to dry in the absence of light in a sterile laminar flow hood. The daily dose of CBD (36 mg/kg bodyweight per day) was calculated based on an adult (around 10 weeks old) male C57BL/6 J mouse eating an average of 0.15 g of standard lab chow per gram bodyweight per day (Bachmanov et al. 2002) and previously generated analytical chemistry data for oral cannabinoid applications (12-day oral treatment period) via chow published elsewhere (Anderson et al. 2019, 2020). The CBD dose (i.e. 36 mg/kg) was chosen based on insights gained from our previous work on other mouse models for neurodegenerative diseases where a range of 5–50 mg/kg of CBD was therapeutically effective to prevent or reverse behavioural impairments (Cheng et al. 2014a, b; Coles et al. 2020; Watt et al. 2020). Reports on food intake in *SOD1*^*G93 A*^ mice are inconsistent and the recording method is often inaccurate (Cocozza et al. 2021; Dupuis et al. 2004; Steyn et al. 2020). Steyn and coworkers used an accurate method in individually housed mice; food intake did not change until at least 11 weeks of age and was increased in ALS transgenic mice from week 16 onwards.

### Behavioural phenotyping

Animals were examined using a number of behavioural tests with relevance to ALS and FTD in line with a previous report from our laboratory (Kreilaus et al. 2020). Test biography and age of the four test cohorts are outlined in Table 1. Motor tests were carried out at the beginning of each week (starting after an initial 7-day treatment period: accelerod on day 1, pole test on day 2), other tests occurring in the same week were performed later in the week (i.e. days 3–5) in line with previous publications (Guerra et al. 2021; Kreilaus et al. 2020). Test protocol specifics are outlined below and follow our established test procedures (Kreilaus et al. 2019; Watt et al. 2020). Bodyweight was recorded weekly. Test equipment was cleaned with 80% ethanol in between test subjects and males were always tested first in the various paradigms before any females were assessed.

**Table 1:** Test biography: The behavioural test order and age [weeks ± 4 days] at testing in male and female *SOD1*^*G93 A*^ (*SOD1*) transgenic and wild type-like (WT) littermates, chronically fed with either a CBD-enriched (CBD) or standard chow (Control) diet. Sample size: WT control males *n* = 9; WT control females *n* = 10; *SOD1* control males *n* = 8; *SOD1* control females *n* = 8; WT CBD males *n* = 7; WT CBD females *n* = 13; *SOD1* CBD males *n* = 11; *SOD1* CBD females *n* = 9. Motor tests were carried out at the beginning of each week (accelerod on day 1, pole test on day 2), other tests occurring in the same week were performed later in the week (i.e. days 3–5). Males were always tested first

Behavioural test	Age [weeks]
Motor function (accelerod, pole test)	11–19, weekly testing
Open field (1st)	12
Prepulse inhibition (1st)	13
Social preference test	14
Fear conditioning	15
Open field (2nd)	18
Prepulse inhibition (2nd)	19

#### Accelerod

To assess motor functions including balance, mice were trained for one day (twice for 5 min) at a fixed speed of 12 rpm on the accelerod apparatus (ENV- 574 M, MED Associates Inc., St Albans, VT, USA). The training was only conducted before the first test (i.e. not before subsequent testing in following weeks). On the test days, mice were placed on the accelerod after which an accelerating program was started (acceleration from 4 to 40 rpm over 270 s and then 30 s at a constant speed of 40 rpm; total test time 5 min). The latency for the mouse to fall from the rotating rod was recorded automatically. The test was performed twice on any test day with an ITI of 1 h. The average latency to fall per day was analysed. The accelerod was performed weekly from 11–19 weeks of age.

#### Pole test

To assess motor functions including grip strength mice were placed facing upwards on a vertical pole (diameter: 1 cm; length: 51 cm) (Karl et al. 2003). The time to turn around, and the time to reach the bottom of the pole was recorded. A 60 s cut-off time was given to mice which did not turn or reach the bottom. This test was performed weekly from 11–19 weeks of age.

#### Open field (OF)

To assess locomotive, explorative, and anxiety-related behaviours, mice were placed into an infrared photobeam controlled OF test chamber (MED Associates Inc., St Albans, USA) for 30 min. The test area (43.2 cm × 43.2 cm) was divided into a central and peripheral zone (MED software coordinates for central zone: 3/3, 3/13, 13/3, 13/13); total distance travelled, and vertical activity were automatically measured. The time spent in the centre zone in the first 10 min of testing and the ratio of centre distance travelled vs total distance travelled were used to identify anxiety-related behaviours. OF testing was performed at 12 and 18 weeks of age.

#### Social preference test (SPT)

The SPT was used to measure social approach behaviour (i.e. sociability) and social recognition memory as previously published (Moy et al. 2004) with minor alterations (Cheng et al. 2014a). The test apparatus consisted of three connected chambers (16.5 cm × 19 cm per chamber): a central chamber with clear Plexiglas dividing walls with square passages (height: 4 cm and width: 4 cm). One circular enclosure (height: 16 cm, width: 8 cm; bars spaced 1 cm apart) was placed into each outer chamber to allow contact between mice but prevent fighting. Fresh bedding was added to all chambers prior to each test trial. Test animals were isolated for 1 h prior to testing in a clean cage with fresh nesting material. Test mice were then allowed to habituate to the apparatus for 5 min before being removed from the test apparatus prior to the sociability trial. For the test of sociability, an unfamiliar social conspecific (i.e. a sex-matched, adult A/JArc mouse), was placed into the circular enclosure of one of the two opponent outer chambers in a quasi-randomised manner before the test mouse was returned to the apparatus and allowed to explore the whole apparatus freely for 10 min. Finally, test animals were observed in a 10 min social recognition test trial in which the test mouse was again removed from the apparatus while a second unfamiliar ‘novel’ opponent A/JArc mouse was placed into the circular enclosure of the previously empty chamber. The test mouse was placed into the apparatus and allowed to explore either the familiar mouse (from the previous trial) or the novel, unfamiliar mouse for another 10 min. ANY-Maze™ tracking software assisted in the manual scoring of the time the test mice spent in the different chambers (i.e. sociability trial) or *nosing* the novel/familial A/JArc mouse (i.e. social recognition trial).

#### Prepulse inhibition (PPI)

PPI was used to test for sensorimotor gating (the attenuation of the startle response by a non-startling stimulus). The PPI protocol was carried out as previously described (Cheng et al. 2014a). The test protocol used a startle pulse of 120 dB (but also tested response to 70 dB and 100 dB), three different prepulse intensities (74, 82 and 86 dB) and inter-stimulus intervals of 32, 64, 128 and 256 ms. Acoustic startle response was calculated as the mean amplitude to all startle trials. Percentage of PPI (%PPI) was calculated as: [mean startle response (120 dB)—PPI response/mean startle response (120 dB)] × 100. PPI was averaged across ISIs to produce a mean % PPI for each prepulse intensity.

#### Fear conditioning (FC)

The conditioning of a fear response results from the association of a previously neutral stimulus (e.g. a tone) with an aversive stimulus (e.g. a foot shock) (Owen et al. 1997). The test was carried out as previously published (Olaya et al. 2018). The FC task occurred over 3 days. On the first day (conditioning trial), mice were placed in a test chamber (NIR-022MD, ENV-005 FPU-M, MED Associates Inc.) with a vanilla scent (Queen™ imitation vanilla essence). After an initial 2 min period, an 80 dB conditioned stimulus (CS) was presented for 30 s co-terminating with a 0.4 mA 2 s foot shock (unconditioned stimulus). The same tone/foot shock pairing occurred again 2 min later. The conditioning session ended 2 min after the second shock, taking a total of 7 min. On the second day of testing (context trial), mice were returned to the same apparatus for 7 min with the vanilla scent cue and no tone. On the third day of testing (cue trial), mice were placed into the apparatus but with an altered environment (i.e. a triangular plastic insert added to the chamber to change its overall shape and scent removed) for 9 min. After 2 min, the CS was continuously presented for 5 min with the test concluding 2 min after termination of the CS presentation (i.e. total cue test time of 9 min). Time spent *freezing* in the conditioning phase as well as the context and cue test was measured using Video-freeze™ (MED Associates Inc.) software version 2.7.2.117 (software settings: threshold = 15). In addition, the *freezing* response to the cue was also assessed by comparing average *freezing* for 2 min prior to cue presentation to the average *freezing* for the 5 min during cue presentation.

### Statistical analysis

Main effects of ‘sex’ and interactions of ‘sex’ with other experimental factors were discovered in behavioural parameters using three-way analysis of variance (ANOVA) as well as four-way repeated measures (RM) ANOVA in line with our previous published work on sex effects in *SOD1*^*G93 A*^ transgenic mice (Kreilaus et al. 2020) (listed in Supplementary Table 1). Thus, all data were split by factor ‘sex’ and three-way RM ANOVA were used to examine the effect of the within-subject factors ‘age’ (weekly testing of the same behaviour), ‘time’ (5-min and 1-min blocks in OF and FC, respectively), ‘cue’ (FC), ‘startle pulse’ and ‘prepulse’ (both PPI) and the between-subject factors 'genotype' and 'treatment'. Follow-up analyses [e.g. two-way or one-way (RM) ANOVA] were run where appropriate. Single sample t-tests were performed in the SPT to determine whether test mice had a preference for the mouse chamber (sociability test) and the novel mouse (social novelty test) (i.e. chamber exploration above 50% chance levels). Differences were considered statistically significant if *p* < 0.05. Data are shown as mean ± SEM. F-values and degrees of freedom are presented for ANOVAs and significant effects of ‘genotype’ are shown in figures and tables as ‘*’ (*p < 0.05, **p < 0.01 and ***p < 0.0001), significant effects of ‘treatment’ as ‘#’ (^#^*p* < 0.05, ^##^*p* < 0.01 and ^###^*p* < 0.0001), ‘treatment’ by ‘genotype’ interactions as ‘^δ^’ (^δ^*p* < 0.05, ^δδ^*p* < 0.01 < 0.01 and ^δδδ^*p* < 0.0001), ‘RM’ by ‘genotype’ interactions as ‘ + ’ (^+^*p* < 0.05, ^++^*p* < 0.01 and ^+++^*p* < 0.0001), ‘RM’ by ‘treatment’ interactions ‘^’ (^^^*p* < 0.05, ^^^^*p* < 0.01 and ^^^^^*p* < 0.0001) and ‘RM’ by ‘genotype’ by ‘treatment’ interactions as ‘^>^’ (^>^*p* < 0.05, ^>>^*p* < 0.01 and ^>>>^*p* < 0.0001). Significant single sample t-test results against chance levels are shown in figures as ‘^γ’^ (^γ^*p* < 0.05, ^γγ^*p* < 0.01, ^γγγ^*p* < 0.0001). Individual data points have been added to figures when appropriate. All analyses were performed in IBM SPSS Statistics v25.

## Results

Several significant main effects of ‘sex’ and interactions with ‘sex’ were identified across all paradigms (provided in Supplementary Table 1). Thus, data were analysed split by sex.

### Bodyweight

**Fig. 1:**
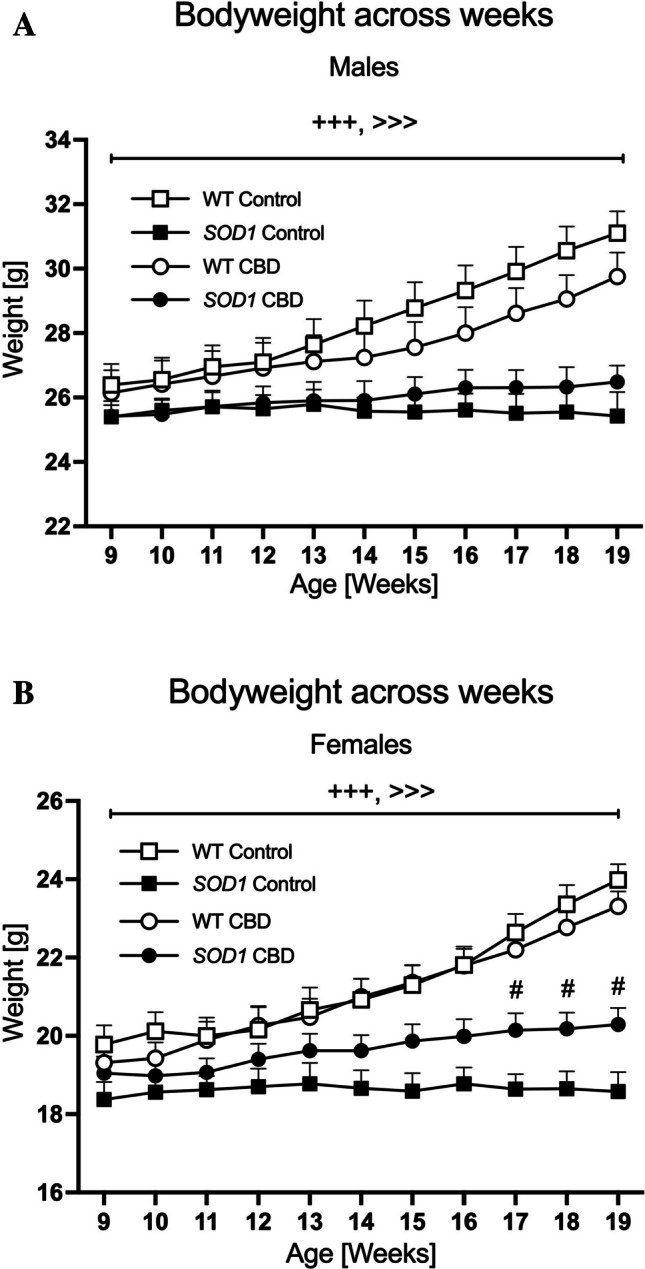
A-B Bodyweight progression: The bodyweight of **A**) male and **B**) female *SOD1*^*G93 A*^ transgenic and wild type-like (WT) littermates, chronically fed with either a CBD-enriched (CBD) or standard chow (Control) diet. Data are shown as mean + SEM. Three-way RM ANOVA ‘age’ by ‘genotype’ interactions are indicated by ^+++^*p* < 0.0001; ‘age’ by ‘genotype’ by ‘treatment’ interactions are indicated by ^>>>^*p* < 0.0001. One-way ANOVA ‘treatment’ effects in *SOD1*^*G93 A*^ females against the corresponding control diet group are indicated by ^#^*p* < 0.05

At the beginning of the experiments, i.e. at 9 weeks of age, *SOD1*^*G93 A*^ and WT males had similar bodyweights [F(1,31) = 2.31; *p* = 0.14]. As expected, three-way RM ANOVA revealed an ‘age’ by ‘genotype’ interaction [F(10,310) = 55.79, *p* < 0.0001] as *SOD1*^*G93 A*^ males gained significantly less weight compared to WT males across the experimental test period (Fig. 1A). Importantly, a significant ‘age’ by ‘genotype’ by ‘treatment’ interaction [F(10,310) = 7.62, *p* < 0.0001] was also detected. Split for ‘genotype’, two-way RM ANOVA found an ‘age’ by ‘treatment’ interaction in *SOD1*^*G93 A*^ males [F(10,170) = 2.35, *p* = 0.013] with CBD-fed *SOD1* mice gaining bodyweight across weeks [RM ANOVA for ‘age’: F(10,100) = 8.19, *p* < 0.0001] while the bodyweight of control-treated *SOD1*^*G93 A*^ did not change [F(10,70) = 0.21, *p* = 0.995]. An ‘age’ by ‘treatment’ interaction was also evident in WT males, however, all mice regardless of treatment increased bodyweight across the testing period (RM ANOVAs for ‘age’ for both treatment groups: *p* < 0.0001, Fig. 1A).

In females, *SOD1*^*G93 A*^ transgenic mice tended to have a lower bodyweight compared to WT mice at 9 weeks of age [F(1,36) = 3.6; *p* = 0.065]. An ‘age’ by ‘genotype’ interaction was evident [F(10,360) = 57.99, *p* < 0.0001] with *SOD1*^*G93 A*^ females gaining significantly less weight over time compared to the WT mice (Fig. 1B). Similar to the findings in males, there was also a significant ‘age’ by ‘genotype’ by ‘treatment’ interaction [F(10,360) = 4.03, *p* < 0.0001]. Split by ‘genotype’, an ‘age’ by ‘treatment’ interaction was found in *SOD1*^*G93 A*^ females [F(10,150) = 3.56, *p* < 0.0001] with CBD-fed *SOD1*^*G93 A*^ female mice gaining weight over time [RM ANOVA for ‘age’: F(10,80) = 19.17, *p* < 0.0001]. This was not evident in control-fed *SOD1*^*G93 A*^ females [F(10,70) = 0.25, *p* = 0.99]. One-way ANOVAs split by week confirmed that CBD-treated *SOD1*^*G93 A*^ had a significantly higher bodyweight compared to control-treated *SOD1*^*G93 A*^ females from week 17 onwards (Fig. 1B). An ‘age’ by ‘treatment’ interaction was also found in WT females, however, all females regardless of treatment increased bodyweight over time (RM ANOVAs for ‘age’ for both treatment groups: *p* < 0.0001, Fig. 1B).

### Motor function

#### Accelerod

**Fig. 2:**
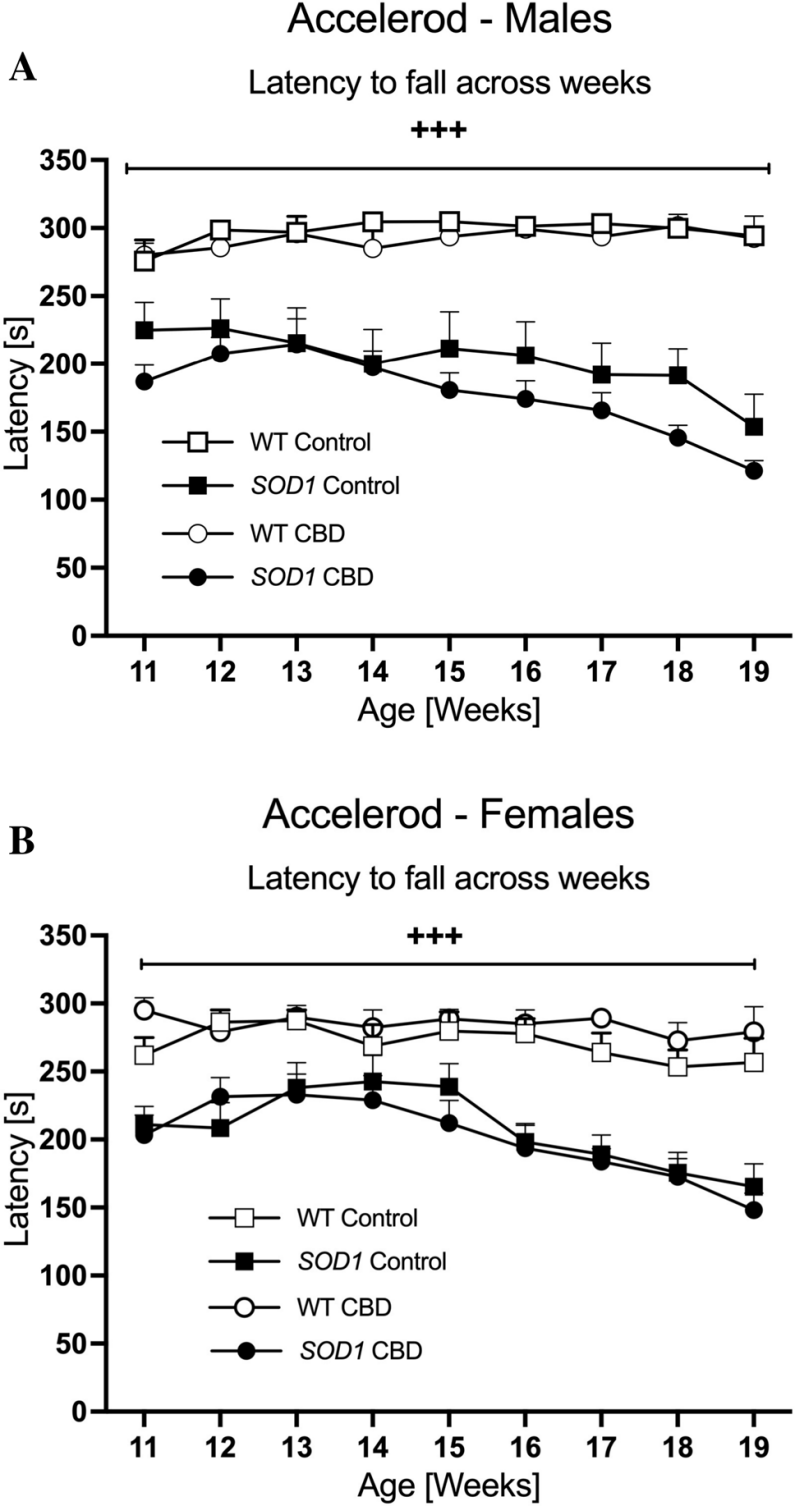
A-B Motor function: The average latency to fall from the accelerod in **A**) male and **B**) female *SOD1*^*G93 A*^ transgenic and wild type-like (WT) littermates, chronically fed with either a CBD-enriched (CBD) or standard chow (Control) diet. Data are shown as mean + SEM. Three-way RM ANOVA ‘age’ by ‘genotype’ interactions are indicated by ^+++^*p* < 0.0001

*SOD1*^*G93 A*^ transgenic males showed poorer motor performance on the accelerod (i.e. latency to fall) starting in the first week of testing [two-way ANOVA for 11-week-old males; ‘genotype’ effect: F(1,31) = 25.75; *p* < 0.0001)]. The three-way RM ANOVA ‘age’ by ‘genotype’ interaction [F(8,248) = 10.68, *p* < 0.0001] indicated that this deficit continued to worsen across the test period and was not affected by CBD treatment (no ‘age’ by ‘genotype’ by ‘treatment’ interaction, *p* > 0.05; Fig. 2A). Indeed, split by ‘genotype’, motor performance significantly worsened over time in *SOD1*^*G93 A*^ males [two-way RM ANOVA for ‘age’: F(8,136) = 12.34, *p* < 0.0001] but improved slightly in WT mice [F(8,112) = 2.07, *p* = 0.044].

Similar to the findings in males, female *SOD1*^*G93 A*^ transgenic mice exhibited poorer motor performance from the start of testing [two-way ANOVA for 11-week-old females: F(1,36) = 36.32; *p* < 0.0001; Fig. 2B] and an ‘age’ by ‘genotype’ interaction was evident [F(8,288) = 6.01, *p* < 0.0001] with CBD having no effect on this interaction (no ‘age’ by ‘genotype’ by ‘treatment’ interaction, *p* > 0.05; Fig. 2B). Split by genotype, *SOD1*^*G93 A*^ transgenic female mice showed worsening motor abilities across age [F(8,120) = 15.29, *p* < 0.0001], whereas the performance of WT females did not significantly change [F(8,168) = 1.14, *p* = 0.34].

#### Pole test

Due to habituation to the test procedure across test weeks, mice lost their natural instinct to climb down to the platform thereby confounding the experimental test results. Thus, data are not discussed in any detail but provided in the Supplementary Figs. 1A-D (statistical analysis included).

### Open field

#### Locomotion and exploration

The OF test was conducted at 12 weeks and 18 weeks of age. In male mice, there were no significant effects or interactions of genotype or treatment on total distance travelled in the 30 min test period in either week (*p* > 0.05 for all comparisons; Table 2). However, a ‘time’ by ‘genotype’ interaction was detected across 5-min blocks at both 12 [F(5,155) = 2.46, *p* = 0.035] and 18 weeks [F(5,155) = 4.53, *p* = 0.001 – Supplementary Figs. 2A/C). When split by 5-min blocks, *SOD1*^*G93 A*^ males travelled less than WT in the first [F(1,31) = 5.08; *p* = 0.031] and second 5-min block [F(1,31) = 4.36; *p* = 0.045] at 18 weeks of age (but not at 12 weeks of age, all *p*’s > 0.05). No significant effects of treatment or genotype (or interactions thereof) were detected for OF *rearing* frequency at 12 weeks of age (*p* > 0.05 for all comparisons, Table 2). However, at 18 weeks of age, a strong trend for reduced *rearing* was evident in *SOD1*^*G93 A*^ transgenic males [F(1,31) = 4.17, *p* = 0.05]. This finding was not affected by CBD treatment (no ‘genotype’ by ‘treatment’ interaction, *p* > 0.05) (Table 2).

**Table 2:** Open field locomotion and exploration: Total distance travelled and *rearing* frequency (Fq) in male and female *SOD1*^*G93 A*^ (*SOD1*) transgenic and wild type-like (WT) littermates, chronically fed with either a CBD-enriched (CBD) or standard chow (Control) diet. Data are shown as mean ± SEM. A two-way ANOVA genotype trend for *rearing* frequency of males in week 18 is indicated by ^ψ^*p* = 0.05

	WT Control	*SOD1* Control	WT CBD	*SOD1* CBD
Males – Week 12 Total Distance [cm]	7336.89 ± 529.70	7683.22 ± 580.27	7303.38 ± 591.26	6724.99 ± 340.49
Males – Week 18 Total Distance [cm]	6048.86 ± 467.75	4985.60 ± 602.81	5076.91 ± 786.60	4802.55 ± 387.62
Males – Week 12 *Rearing* Fq [n]	81.89 ± 7.36	86.13 ± 8.96	87.71 ± 11.0	67.36 ± 6.77
Males – Week 18 *Rearing* Fq [n] ^ψ^	62.78 ± 7.23	52.38 ± 12.34	59.86 ± 9.98	39.73 ± 5.98
Females – Week 12 Total Distance [cm]	6714.28 ± 463.38	6475.46 ± 329.76	6984.45 ± 488.36	6647.03 ± 331.27
Females – Week 18 Total Distance [cm]	5272.41 ± 856.09	5238.75 ± 756.89	4731.66 ± 360.35	4458.41 ± 531.49
Females – Week 12 *Rearing* Fq [n]	60.70 ± 6.86	38.38 ± 6.63	51.46 ± 5.35	45.44 ± 6.17
Females – Week 18 *Rearing* Fq [n]	52.5 ± 6.78	41.25 ± 7.21	40.38 ± 4.9	32.11 ± 6.47

In female mice, neither OF locomotion nor exploration were different across experimental conditions at 12 and 18 weeks (*p* > 0.05 for all comparisons; Table 2) and no interactions across time were found either (*p* > 0.05 for all comparisons – Supplementary Figs. 2B/D).

#### Anxiety-related behaviours

**Fig. 3:**
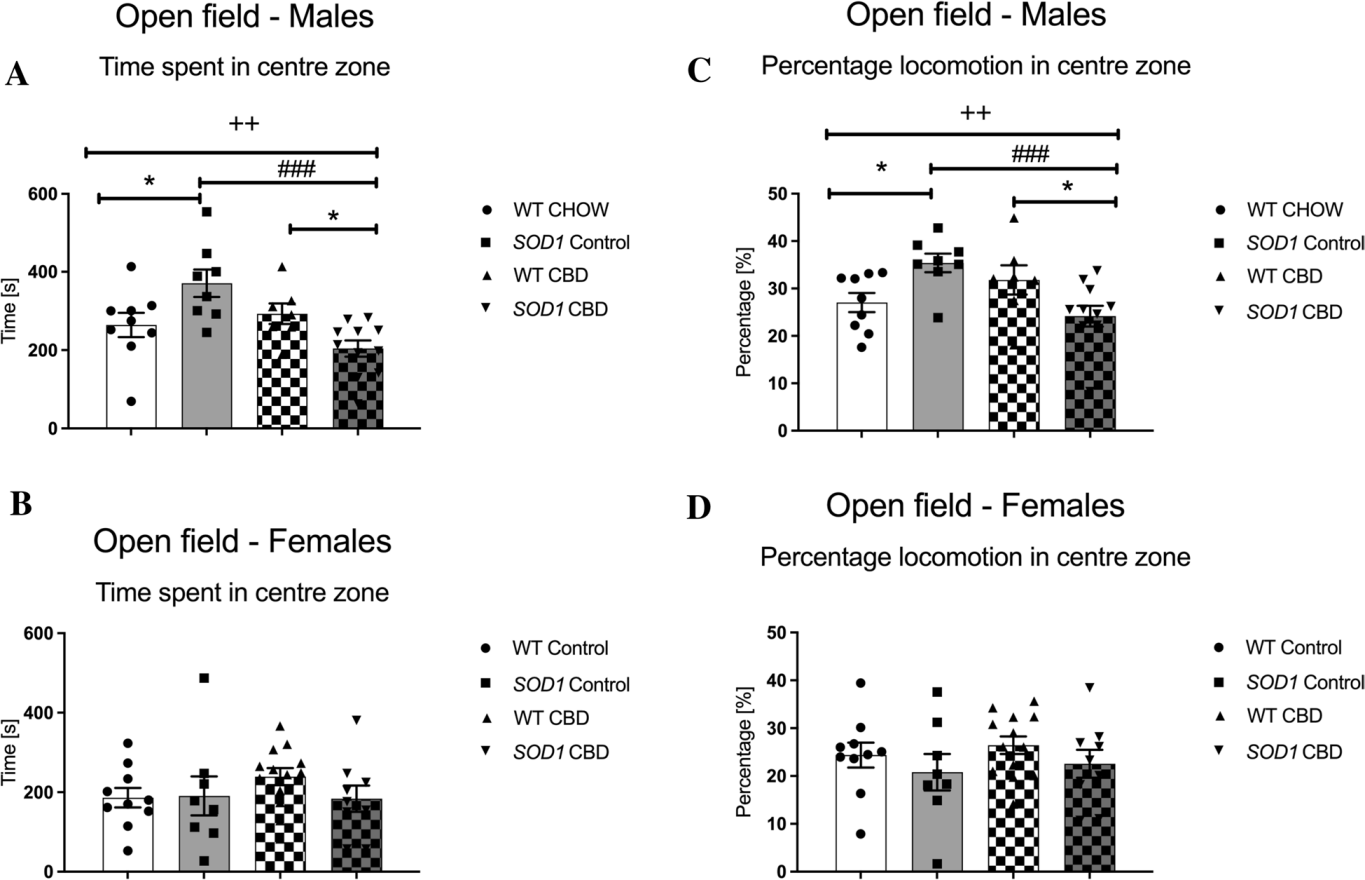
A-D Anxiety-relevant behaviours in the open field: **A-B**) Time spent [s] and **C-D**) percentage locomotion (i.e. distance travelled) [%] in the centre zone in **A/C**) male and **B/D**) female *SOD1*^*G93 A*^ transgenic and wild type-like (WT) littermates (at 12 weeks of age), chronically fed with either a CBD-enriched (CBD) or standard chow (Control) diet. Data are shown as mean ± SEM. Two-way ANOVA ‘treatment’ by ‘genotype’ interactions are indicated by ^++^*p* < 0.01. One-way ANOVA effects of ‘treatment’ are indicated by ^###^*p* < 0.0001 and effects of ‘genotype’ are indicated by *p < 0.05

In 12-week-old males, ‘genotype’ by ‘treatment’ interactions were evident for both the time spent [F(1,31) = 11.74, *p* = 0.002] as well as the percentage locomotion in the centre zone [F(1,31) = 13.2, *p* = 0.001]. Split by ‘genotype’, chronic CBD treatment reduced time spent [F(1,17) = 18.85, *p* < 0.0001] and percentage locomotion [F(1,17) = 18.55, *p* < 0.0001] in *SOD1*^*G93 A*^ mice when compared to control diet-fed *SOD1*^*G93 A*^ mice, while CBD diet had no significant effect in WT mice (all *p*’s > 0.05 – Fig. 3A/C). Interestingly, split by ‘treatment’, *SOD1*^*G93 A*^ males were less anxious than WT males when fed control CHOW diet [time: F(1,15) = 5.22, *p* = 0.037 – percentage locomotion: F(1,15) = 6.45, *p* = 0.023], while CBD-treated *SOD1*^*G93 A*^ males were more anxious compared to CBD-treated WT males [time: F(1,15) = 7.08, *p* = 0.017 – percentage locomotion: F(1,16) = 6.87, *p* = 0.018; Fig. 3A/C). At 18 weeks of age, no significant effects of genotype or treatment (or interactions thereof) were found (all *p’s* > 0.05; Supplementary Table 2).

In female mice, no significant main effects or interactions for the time spent and the percentage locomotion in the centre zone were found at either age tested (all *p’s* > 0.05; for 12-week-old females: Fig. 3B/D – for 18-week-old females: Supplementary Table 2).

### Sociability and social recognition memory

#### Sociability

**Fig. 4:**
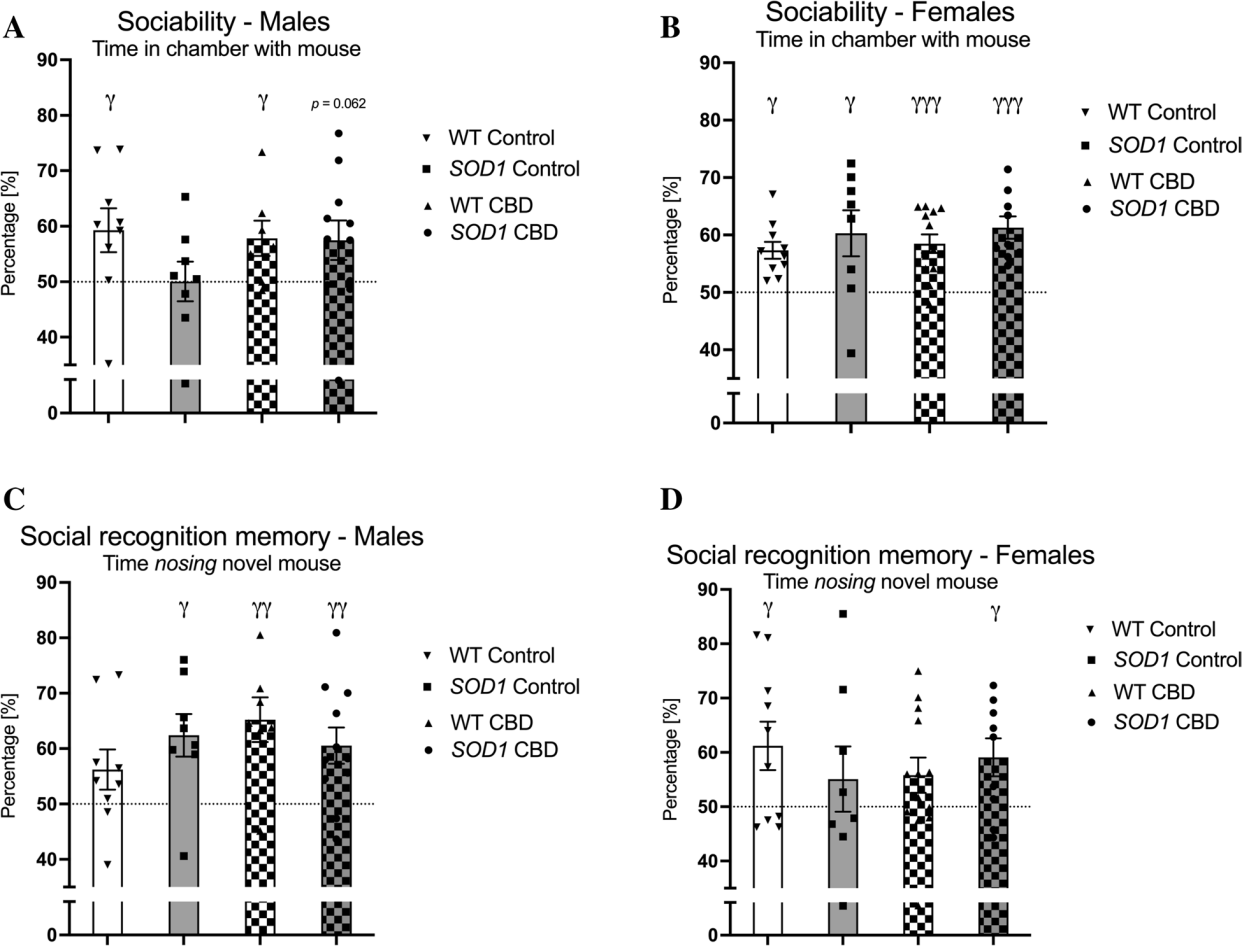
A-D Sociability and social recognition memory: **A-B**) Percent time spent in a chamber with a mouse [%] and **C-D**) percent time spent *nosing* a novel mouse [%] in **A/C**) male and **B/D**) female *SOD1*^*G93 A*^ transgenic and wild type-like (WT) littermates, chronically fed with either a CBD-enriched (CBD) or standard chow (Control) diet. Data are shown as mean ± SEM. Single sample t-test vs. 50% chance level are indicated by ^γ^*p* < 0.05, ^γγ^* p* < 0.01, ^γγγ^* p* < 0.0001. There was also a trend in CBD-treated *SOD1*^*G93 A*^ transgenic males to have intact sociability (*p* = 0.062; Fig. 4A)

In male mice, single sample t-tests indicated that only WT mice showed a significant preference for the mouse chamber regardless of treatment [WT Control: t(8) = 2.36, *p* = 0.046, WT CBD: t(6) = 2.46, *p* = 0.049] although there was also a trend in CBD-treated *SOD1*^*G93 A*^ transgenic males to have intact sociability [*SOD1*^*G93 A*^ Control: t(7) = 0.01, *p* = 0.99, *SOD1*^*G93 A*^ CBD: t(10) = 2.1, *p* = 0.062 – Fig. 4A]. In females, all groups spent significantly more than 50% of the time in the chamber with the test mouse [WT Control: t(9) = 5.02, *p* = 0.001, WT CBD: t(12) = 5.25, *p* < 0.0001, *SOD1*^*G93 A*^ Control: t(7) = 2.57, *p* = 0.037, *SOD1*^*G93 A*^ CBD: t(8) = 5.79, *p* < 0.0001 – Fig. 4B].

#### Social recognition memory

In males, all experimental groups except WT mice fed a standard chow diet spent significantly more than 50% of the time *nosing* the novel mouse [WT Control: t(8) = 1.71, *p* = 0.13, WT CBD: t(6) = 3.77, *p* = 0.009, *SOD1*^*G93 A*^ Control: t (7) = 3.23, *p* = 0.014, *SOD1*^*G93 A*^ CBD: t(10) = 3.22, *p* = 0.009 – Fig. 4C]. In females, only WT fed a standard chow and CBD-treated *SOD1*^*G93 A*^ transgenic females exhibited a significant preference for the novel mouse [WT Control: t(9) = 2.51, *p* = 0.033, WT CBD: t (12) = 1.79, *p* = 0.099, *SOD1*^*G93 A*^ Control: t(7) = 0.85, *p* = 0.43, *SOD1*^*G93 A*^ CBD: t(8) = 2.6, *p* = 0.032 – Fig. 4D].

### Sensorimotor gating

#### Acoustic startle response

**Fig. 5:**
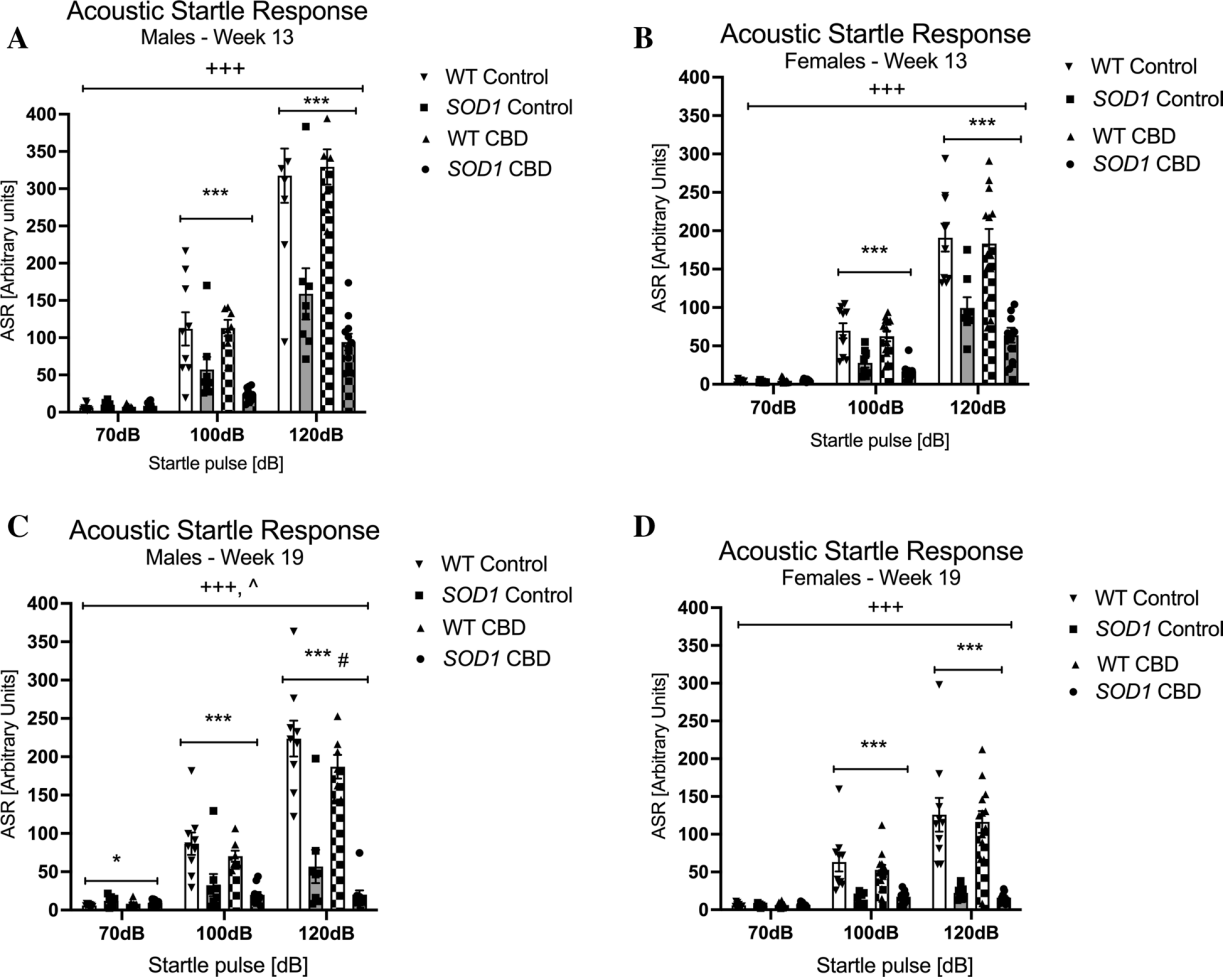
A-D Acoustic startle response: Acoustic startle response at **A-B**) 13 weeks and **C-D**) 19 weeks of age in **A/C**) male and **B/D**) female *SOD1*^*G93 A*^ transgenic and wild type-like (WT) littermates, chronically fed with either a CBD-enriched (CBD) or standard chow (Control) diet. Data are shown as mean ± SEM. Three-way RM ANOVA ‘startle pulse’ by ‘genotype’ interactions are indicated by ^+++^*p* < 0.0001, ‘startle pulse’ by ‘treatment’ interactions are indicated by ^*p* < 0.05. Two-way ANOVA ‘treatment’ effects per startle pulse intensity are indicated by ^#^*p* < 0.05, ‘genotype’ effects are indicated by *p < 0.05 and ***p < 0.0001

In male mice, *SOD1*^*G93 A*^ had an overall lower startle response at 13 weeks of age [three-way RM ANOVA for ‘genotype’: F(1,31) = 44.73; *p* < 0.0001 – Fig. 5A]. A ‘startle pulse’ by ‘genotype’ interaction was also found [F(2,62) = 45.13, *p* < 0.0001]. Split by ‘genotype’, both genotypes responded to increasing startle pulses [WT: F(2, 30) = 129.15; *p* < 0.0001—*SOD1*^*G93 A*^: F(2,36) = 111.56; *p* < 0.0001]. The startle response was weaker in *SOD1*^*G93 A*^ mice compared to WT animals across treatment conditions at both 100 dB and 120 dB as indicated by two-way ANOVA main effects of ‘genotype’ (split by ‘startle pulse’) (Fig. 5A). No effects of treatment or interactions with genotype or startle pulse intensity were detected at 13 weeks of age (all *p’s* > 0.05). Similarly, at 19 weeks of age, *SOD1*^*G93 A*^ males had an overall lower startle response when compared to WT [F(1,31) = 65.02; *p* < 0.0001 – Fig. 5C]. A ‘startle pulse’ by ‘genotype’ interaction was also found [F(2,62) = 84.42, *p* < 0.0001]. Split by ‘genotype’, both genotypes responded to increasing startle pulses [WT: F(2, 30) = 152.14; *p* < 0.0001—*SOD1*^*G93 A*^: F(2,36) = 5.21; *p* = 0.01]. Again, this response was weaker in *SOD1*^*G93 A*^ mice at both 100 dB and 120 dB as indicated by two-way ANOVA main effects of ‘genotype’ (split by ‘startle pulse’) (Fig. 5C). In addition, a small but significant elevation of ASR was detected in *SOD1*^*G93 A*^ mice at 70 dB (background noise). Also, a ‘startle pulse’ by ‘treatment’ interaction [F(2, 62) = 3.80, *p* = 0.028] was evident and suggested that CBD attenuated the response to increasing startle pulses compared to standard chow-fed males regardless of genotype (no ‘genotype’ by ‘treatment’ by ‘startle pulse’ interaction, *p* > 0.05; Fig. 5C). Split by treatment, the startle response still increased with increasing dB levels in both treatment groups [Control: F(2, 30) = 74.72; *p* < 0.0001—CBD: F(2,32) = 241.2; *p* < 0.0001] but this response was weaker in mice treated with CBD as indicated by a main effect of ‘treatment’ at 120 dB (Fig. 5C).

In females, *SOD1*^*G93 A*^ mice exhibited a lower ASR compared to WT females at 13 weeks of age [three-way RM ANOVA for ‘genotype’: F(1,36) = 42.78; *p* < 0.0001] and 19 weeks of age [F(1,36) = 43.29; *p* < 0.0001 – Fig. 5B/D]. A ‘startle pulse’ by ‘genotype’ interaction was also evident [at 13 weeks: F(2, 72) = 31.28, *p* < 0.0001 – at 19 weeks: F(2, 72) = 40.66, *p* < 0.0001; Fig. 5B/D]. Both genotypes displayed increasing startle to increasing startle pulses at 13 weeks [WT: F(2,44) = 171.95; *p* < 0.0001—*SOD1*^*G93 A*^: F(2,32) = 57.43; *p* < 0.0001] and 19 weeks [WT: F(2,44) = 75.41; *p* < 0.0001—*SOD1*^*G93 A*^: F(2,32) = 18.97; *p* < 0.0001], but this response was weaker in *SOD1*^*G93 A*^ as indicated by reduced ASR compared to WT females at 100 dB and 120 dB startle pulses in both test weeks (Fig. 5B/D). CBD did not affect these interactions (i.e. no ‘startle pulse’ by ‘genotype’ by ‘treatment’ interactions, all *p*’s > 0.05).

#### Prepulse inhibition

**Fig. 6:**
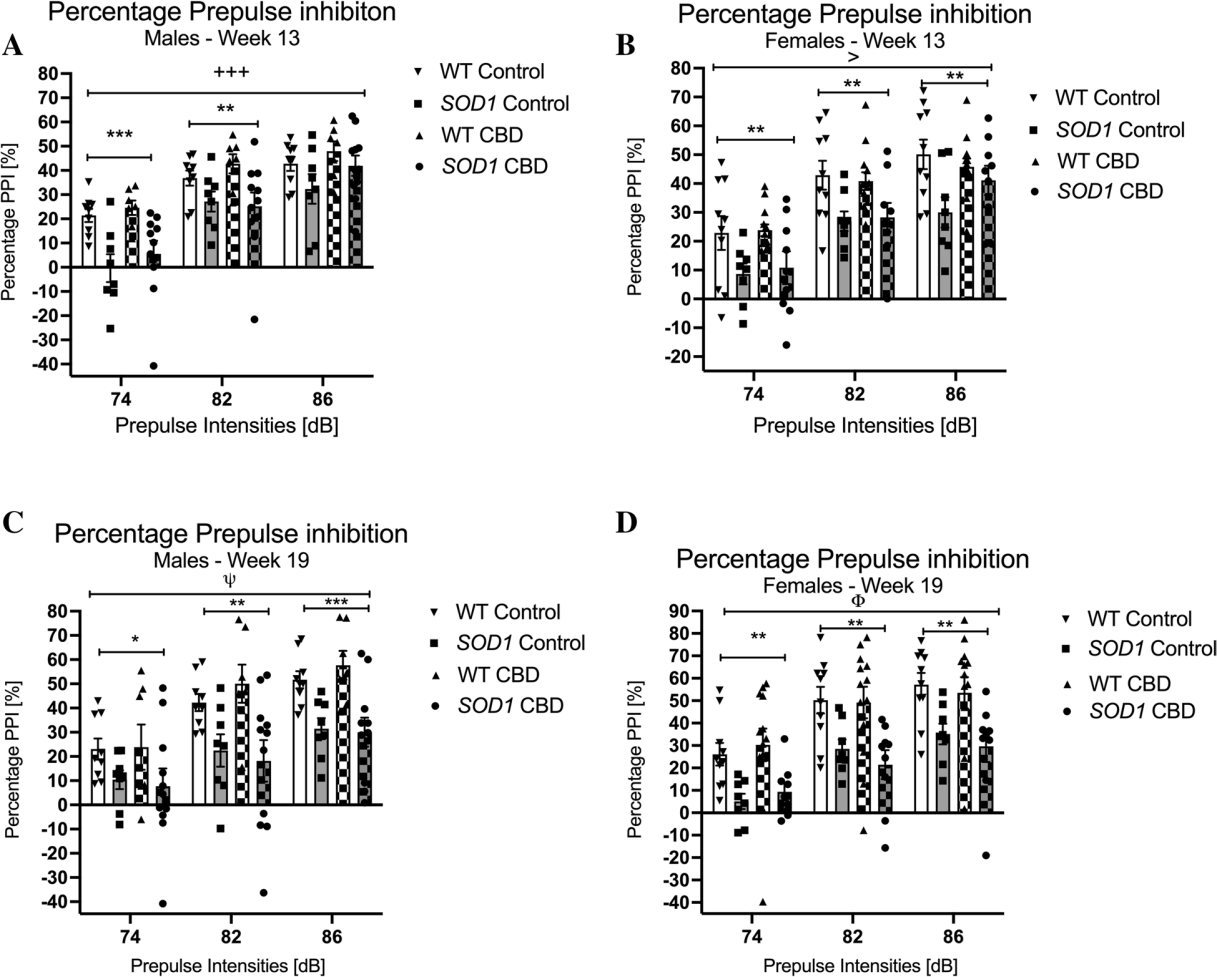
A-D Prepulse inhibition: Prepulse inhibition [%] at 74, 82 and 86 dB prepulse intensities averaged across inter stimulus intervals at **A-B**) 13 weeks and **C-D**) 19 weeks of age in **A/C**) male and **B/D**) female *SOD1*^*G93 A*^ transgenic and wild type-like (WT) littermates, chronically fed with either a CBD-enriched (CBD) or standard chow (Control) diet. Data are shown as mean ± SEM. Three-way RM ANOVA ‘prepulse’ by ‘genotype’ interactions are indicated by ^+^*p* < 0.05, ^+++^*p* < 0.0001 (^ψ^*p* = 0.062 trend), ‘prepulse pulse’ by ‘genotype’ by ‘treatment’ interactions are indicated by ^>^*p* < 0.05 and a trend for a ‘prepulse’ by ‘treatment’ interaction is shown by ^Φ^*p* = 0.063. Two-way ANOVA ‘genotype’ effects at each prepulse intensity are indicated by *p < 0.05, **p < 0.01 and ***p < 0.0001

At 13 weeks of age, *SOD1*^*G93 A*^ males had an overall lower %PPI when compared to WT mice [three-way RM ANOVA for ‘genotype’: F(1,31) = 10.46; *p* = 0.003], and a ‘prepulse’ by ‘genotype’ interaction [F(2,62) = 8.83, *p* < 0.0001] was also found (Fig. 6A). Split by ‘genotype’, all male mice responded to increasing prepulse intensities with increasing %PPI [‘prepulse’ for WT: F(2, 30) = 133.94; *p* < 0.0001—*SOD1*^*G93 A*^: F(2,36) = 102.99; *p* < 0.0001], however, split by ‘prepulse’, significantly reduced %PPI of *SOD1*^*G93 A*^ males at 74 dB and 82 dB prepulse intensities suggested a sensorimotor gating deficit. At 19 weeks of age, *SOD1*^*G93 A*^ males had an overall lower %PPI when compared to WT mice [F(1,31) = 13.61; *p* = 0.001], with significant differences detected at 74 dB, 82 dB and 86 dB prepulse intensities (Fig. 6C). In addition, a trend for a ‘prepulse’ by ‘genotype’ interaction was detected [F(2, 62) = 2.9, *p* = 0.062; Fig. 6C], but mice of both genotypes responded to increasing prepulse intensities with increasing %PPI [split by ‘genotype’ for WT: F(2, 30) = 66.70; *p* < 0.0001—*SOD1*^*G93 A*^: F(2,36) = 15.86; *p* < 0.0001 – Fig. 6C]. No effects or interactions of CBD treatment were evident for either week (*p* > 0.05 for all comparisons).

*SOD1*^*G93 A*^ females at 13 weeks of age had an overall lower %PPI compared to WT mice [F(1,36) = 10.93; *p* = 0.002], and this difference was evident at 74 dB, 82 dB and 86 dB prepulse intensities (Fig. 6B). All mice responded to increasing prepulses with increasing %PPI [F(2,72) = 169.53; *p* < 0.0001 – no ‘prepulse’ by ‘genotype’ interaction, *p* > 0.05]. Importantly, a significant ‘prepulse’ by ‘genotype’ by ‘treatment’ interaction was evident [F(2, 72) = 3.66, *p* = 0.031] as CBD treatment appeared to correct the %PPI deficit in *SOD1*^*G93 A*^ mice across prepulse intensities (Fig. 6B). However, when looking at individual ‘prepulse’ intensities, no ‘genotype’ by ‘treatment’ interactions were detected at any particular prepulse intensity (all *p’s* > 0.05). Significantly reduced %PPI of *SOD1*^*G93 A*^ mice at all three prepulse intensities suggested a sensorimotor gating deficit. At 19 weeks, *SOD1*^*G93 A*^ females had an overall lower %PPI when compared to WT mice [F(1,36) = 15.22; *p* < 0.0001], and this difference was evident at 74, 82 and 86 dB prepulse intensities (Fig. 6D). All mice responded to increasing prepulses with increasing %PPI [F(2,72) = 87.44; *p* < 0.0001 – no ‘prepulse’ by ‘genotype’ interaction, *p* > 0.05]. A trend for a ‘prepulse’ by ‘treatment’ interaction was found [F(2, 72) = 2.88, *p* = 0.063 – Fig. 6D]. No other effects or interactions of CBD treatment were evident (*p* > 0.05 for all comparisons).

### Fear-associated memory

Baseline *freezing* (first two min of conditioning test) of male and female mice was not significantly different across experimental groups (no significant main effects or ‘genotype’ by ‘treatment’ interactions: *p* > 0.05 for all comparisons; data not shown).

#### Context

No differences were found in total duration of context *freezing* in male mice regardless of genotype or treatment (or interactions thereof; *p* > 0.05 for all comparisons – Table 3). In female mice, a significant main effect of a ‘genotype’ was found [F(1,36) = 5.8, *p* = 0.02] as *SOD1*^*G93 A*^ transgenic females *froze* significantly longer in the context test than WT mice (Table 3). This difference was not affected by CBD (no ‘genotype’ by ‘treatment’ interaction: *p* > 0.05).

**Table 3:** Fear-associated memory (i.e. context *freezing*): Total *freezing* time in the context trial of the fear conditioning test. Data from male and female *SOD1*^*G93 A*^ (*SOD1*) transgenic and wild type-like (WT) littermates, chronically fed with either a CBD-enriched (CBD) or standard chow (Control) diet are shown as mean ± SEM. A two-way ANOVA genotype effect is indicated by *p < 0.05

	WT Control	*SOD1* Control	WT CBD	*SOD1* CBD
Males *Freezing* [s]	77.73 ± 16.42	62.36 ± 20.40	94.81 ± 17.91	101.27 ± 11.52
Females *Freezing* [s] *	77.45 ± 22.82	155.53 ± 34.53	94.47 ± 17.76	134.6 ± 25.62

#### Cue

**Fig. 7:**
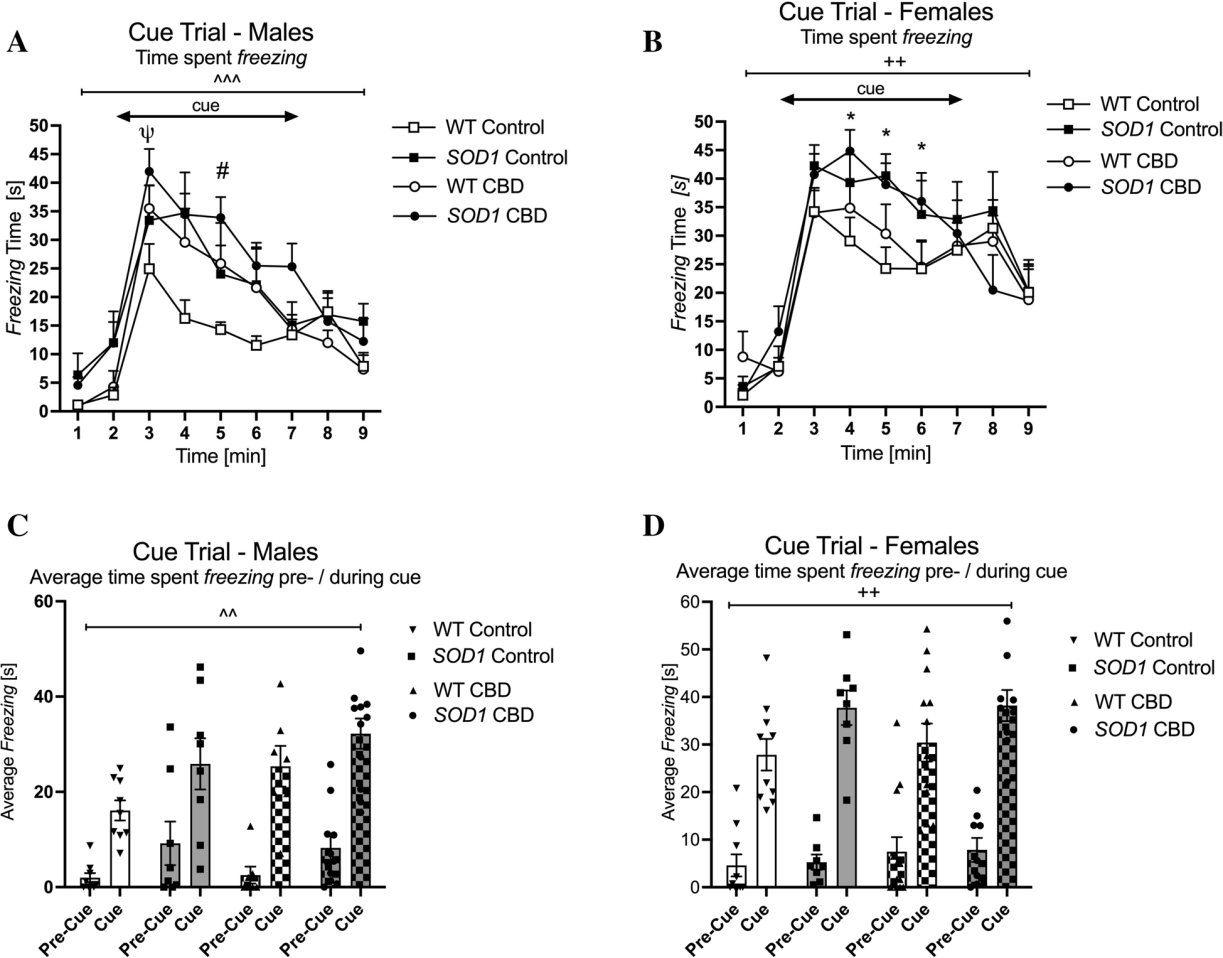
A-D Fear conditioning: Fear-associated *freezing* in the cue trial **A-B**) *freezing* time per minute [s], and **C-D**) average *freezing* pre-cue and during cue [s]. Testing was conducted in **A/C**) male and **B/D**) female *SOD1*^*G93 A*^ transgenic and wild type-like (WT) littermates, chronically fed with either a CBD-enriched (CBD) or standard chow (Control) diet. Data are shown as mean + SEM. Three-way RM ANOVA interactions between ‘treatment’ and either ‘time’ (**7 A**) or ‘cue’ (**7 C**) are indicated by ^^*p* < 0.01 and ^^^ *p* < 0.0001. Interactions between ‘genotype’ and either ‘time’ (**7B**) or ‘cue’ (**7 C**) are shown by ^++^*p* < 0.01 and ^+++^*p* < 0.0001. Two-way ANOVA ‘treatment’ effects across genotypes are indicated by ^#^*p* < 0.05 (^ψ^*p* = 0.05 trend), ‘genotype’ effects across treatment are indicted by *p < 0.05

Male *SOD1*^*G93 A*^ mice displayed higher levels of total cue *freezing* than WT mice [main effect of ‘genotype’: F(1,31) = 4.99, *p* = 0.033—no ‘genotype’ by ‘treatment’ interaction: *p* > 0.05; Fig. 7A]. In addition, a ‘time’ by ‘treatment’ interaction was detected [F(8,248) = 3.67, *p* < 0.0001] as CBD-treated males showed a stronger *freezing* response to the cue compared to males on control diet. Indeed, split by ‘time’, CBD-treated males exhibited increased cue *freezing* at the 3rd and 5th minute of the test compared to mice fed a standard chow (Fig. 7A—no ‘genotype’ by ‘treatment’ interactions for any test minute, *p* > 0.05 for all comparisons). Similarly, comparing the average *freezing* time pre-cue with during cue, three-way RM ANOVA revealed a ‘cue’ by ‘treatment’ interaction [F(1,31) = 11.04, *p* = 0.002]. Split by treatment, all mice showed an increase in *freezing* in response to the cue [Control: F(1,15) = 80.18; *p* < 0.0001, CBD: F(1,16) = 191.72; *p* < 0.0001], however, CBD-treated males had a greater percentage increase in their *freezing* response to the cue compared to males on control diet (Control: 285% increase—CBD: 391% increase—Fig. 7C).

In females, three-way RM ANOVA found a significant ‘time’ by ‘genotype’ interaction for cue *freezing* [F(8, 288) = 2.9, *p* = 0.004] (Fig. 7B). Split by ‘time’, *SOD1*^*G93 A*^ females regardless of treatment *froze* more than WT at the 4th, 5th, and 6th minute (no ‘treatment’ by genotype’ interactions: *p’s* > 0.05; Fig. 7B). Similarly, comparing the average *freezing* time pre-cue with during cue, a ‘cue’ by ‘genotype’ interaction was found [F(1, 36) = 8.8, *p* = 0.005; Fig. 7D]. Split by genotype, all mice responded to the cue [WT: F(1,21) = 193.92; *p* < 0.0001, *SOD1*^*G93 A*^: F(1,15) = 174.81; *p* < 0.0001], however, *SOD1*^*G93 A*^ females showed a greater percentage increase in their *freezing* response to the cue compared to WT females (WT: 371%, *SOD1*^*G93 A*^ 473%—Fig. 7D). No interactions or effects of CBD treatment were detected in female mice.

## Discussion

Our study investigating dietary CBD supplementation in the *SOD1*^*G93 A*^ mouse model of ALS has identified several beneficial effects of CBD, many that were specific to sex, genotype or age/treatment duration. Most importantly, CBD treatment ameliorated the bodyweight loss phenotype in male and, more prominently, female *SOD1*^*G93 A*^ mice, tended to reinstate sociability in *SOD1*^*G93 A*^ males, strengthened social recognition memory in *SOD1*^*G93 A*^ females, and improved the PPI response in younger *SOD1*^*G93 A*^ females, in particular at higher prepulse intensities. CBD treatment reversed the anxiolytic-like OF phenotype of 12-week-old male *SOD1*^*G93 A*^ mice. The phytocannabinoid decreased ASR in both WT and *SOD1*^*G93 A*^ males in week 19 and also improved cued fear conditioning in all male mice. Independent of CBD treatment, all *SOD1*^*G93 A*^ mice gained less bodyweight across the experimental period, exhibited poorer motor performance and had lower ASR and %PPI compared to their WT littermates. Sex-specifically, *SOD1*^*G93 A*^ males showed more cue *freezing* than WT males, whereas *SOD1*^*G93 A*^ females displayed stronger *freezing* responses than WT females in both context and cue FC testing.

The *SOD1*^*G93 A*^ mouse model is characterised by a deficit in bodyweight gain during disease development, which was evident in both male and female control-treated *SOD1*^*G93 A*^ mice in the current study. Importantly, CBD treatment attenuated this phenotype, resulting in a small but significant positive change in bodyweight across the study in both male and more prominently female *SOD1*^*G93 A*^ mice. It is important to note that we observed similar motor activity across treatment conditions, a factor that could otherwise have confounded CBD effects on bodyweight development. Hypermetabolism is commonly observed in *SOD1*^*G93 A*^ transgenic mice as well as human ALS patients and both exhibit higher resting energy expenditure (Bouteloup et al. 2009; Dupuis et al. 2004). Mitochondrial dysfunction is one mechanism suggested to contribute to this phenotype, with abnormalities in mitochondrial morphology observed in both human ALS and *SOD1*^*G93 A*^ mice (Higgins et al. 2003; Sasaki and Iwata 2007). Interestingly, a faster reduction of body mass index (BMI) in ALS patients prior to disease onset is correlated with shorter survival (Shimizu et al. 2012) and more rapid BMI decline in early disease stages is associated with accelerated functional deterioration (Nakayama et al. 2019). Interestingly, other preclinical studies into new therapeutics for ALS utilising the *SOD1*^*G93 A*^ mouse also found that attenuated bodyweight-loss is accompanied by increased survival rates, for example in studies targeting energy metabolism, oxidative stress, or inflammation (Kiaei et al. 2006; Petri et al. 2006). A previous study testing the cannabinoid receptor 2 (CB_2_) agonist AM1241 also reported increased survival of *SOD1*^*G93 A*^ transgenic mice (bodyweight was not reported) (Shoemaker et al. 2007). CBD has a low affinity for the CB_2_ receptor and has been proposed to be a negative allosteric modulator of this receptor (Martínez-Pinilla et al. 2017). This suggests that CBD’s effects on bodyweight may be mediated through its activity on different receptors [such as serotonin 1 A receptor (5-HT_1 A_)] or CBD’s anti-inflammatory (Mori et al. 2017) and antioxidant properties (Hampson et al. 1998).

Motor performance was poorer in *SOD1*^*G93 A*^ transgenic mice compared to WT at 11 weeks of age, and CBD treatment did not alter the motor phenotype in either genotype. The disease course is rapid in *SOD1*^*G93 A*^ mice, and although overt motor deficits on the accelerod typically appear at 12–16 weeks, subtle motor deficits (including lower muscle force [Saxena et al. 2009), grip strength (Ligon et al. 2005) and locomotion velocity (Hayworth and Gonzalez-Lima 2009)] are already evident at 4–7 weeks of age. In line with these behavioural changes, biomolecular changes [e.g. elevated neuroinflammation (Gifondorwa et al. 2012; Gould et al. 2006)] and defective energy metabolism (Browne et al. 2006) have been reported in *SOD1*^*G93 A*^ mice as early as 4–6 weeks of age. Thus, future studies should consider beginning treatment earlier than at 10 weeks of age to observe the full potential of new treatment candidates including CBD for mild disease stages. Early intervention is clinically relevant as it most closely represents the early diagnostic period in patients.

Motor activity was not different across experimental cohorts but *SOD1*^*G93 A*^ males habituated to the OF arena differently compared to WT at both 12 and 18 weeks of age. This appeared to be mostly driven by lower locomotor activity of the transgenic mice during the first 10 min of OF testing and could be due to the progressively worsening motor impairment (including reduced *rearing*, which was not evident in females) affecting ambulatory velocity. Differences in anxiety behaviour can also modify habituation but this is unlikely for the current study as the changes to OF habituation of *SOD1*^*G93 A*^ mice was strongest at 18 weeks of age, at which time no pronounced anxiety phenotype was observed in transgenic mice. Interestingly, our previous study in the same model at around 11 weeks of age did not identify altered habituation to the OF arena (Kreilaus et al. 2020).

*SOD1*^*G93 A*^ males of the current study displayed an anxiolytic-like phenotype at 12 weeks of age which is in line with earlier studies in 8- and 11-week-old *SOD1*^*G93 A*^ males (Kreilaus et al. 2020; Quarta et al. 2015). Interestingly, this phenotype was age-dependent, as it was absent in 18-week-old transgenic mice—a novel finding for this mouse model. Variation in findings across studies may be related to different test biographies, with handling and testing of transgenic mice in the current study occurring prior to the first OF test whereas earlier studies used test-naïve animals (Kreilaus et al. 2020; Quarta et al. 2015). Indeed, it is well established that repeated testing and handling (Võikar et al. 2004) and the handling method itself (Gouveia and Hurst 2013) can alter mouse behaviour and anxiety phenotypes, and therefore must be considered when comparisons are made across studies. Interestingly, our long-term CBD treatment had anxiogenic-like effects in young male *SOD1*^*G93 A*^ mice specifically thereby reversing the ALS model phenotype evident under control treatment conditions. Previous studies evaluating CBD effects in the OF and the elevated plus maze have reported either no anxiety effects (similar to what we found in WT mice in the current study) or anxiolytic-like effects which were dependent on dose and length of treatment (i.e. acute vs chronic treatments) (Guimarães et al. 1990; Long et al. 2012; Watt et al. 2020). Further comprehensive studies are required to clarify the effects of various doses of acute and chronic oral CBD treatment on anxiety domains in this ALS mouse model as the therapeutic relevance of the current finding is unclear at this stage.

*SOD1*^*G93 A*^ males showed a sociability deficit similar to that observed previously in this mouse model (Kreilaus et al. 2020). It has been reported that chronic CBD administration can improve sociability in mice and rats (Long et al. 2012; Osborne and Solowij 2017). Indeed, we found a trend for a similar CBD effect in our study suggesting a moderate beneficial effect of oral CBD treatment on this behavioural impairment. The finding raises the question if higher dosing or earlier initiation of CBD treatment could have resulted in more robust socio-positive effects of CBD in this ALS mouse model. When testing social recognition memory in males, WT control-treated mice were the only group that did not develop a preference for the novel mouse. In females, control-treated *SOD1*^*G93 A*^ had no preference for the novel mouse, whereas CBD treatment re-installed that preference in ALS transgenic females. The effect of CBD in female *SOD1*^*G93 A*^ mice is consistent with CBD restoring social recognition memory in past neurodegenerative disease models (Cheng et al. 2014a; Watt et al. 2020). However, as WT CBD-treated mice failed to show a preference for the novel mouse, these results should be interpreted with caution. We have previously not identified a negative impact of CBD on social recognition memory (Cheng et al. 2014a; Watt et al. 2020). The sociability deficit of male SOD1^G93 A^ mice could be related to a reduced motivational drive for social interaction resulting in the avoidance of social encounters where possible. Importantly, sociability and social recognition memory can be distinct from each other as is evident in SOD1^G93 A^ transgenic males of the current study. Indeed, adult C57BL/6J mice exhibited an impaired sociability but intact social recognition memory in one of the first studies evaluating this test paradigm (Moy et al. 2004) – similar has been described for other inbred strains (Moy et al. 2008). The drive for social encounters can be low without affecting the ability to differentiate between novel and familiar social stimuli or impairing social recognition memory more generally. Similar phenotypes have been reported in the context of autism spectrum disorder and schizophrenia [for example: (Moy et al. 2009)] suggesting those animals can process social information but have reduced interest in social interaction although it should be stated that often sociability and social novelty preference are both affected.

The ASR was lower in both male and female *SOD1*^*G93 A*^ mice at 13 weeks of age, and this deficit was more pronounced at 19 weeks of age. Although it is likely that a hearing deficit may contribute to this lower startle response, our current data and previous studies (Guerra et al. 2021; Kreilaus et al. 2020) found that both male and female *SOD1*^*G93 A*^ transgenic mice respond to an auditory tone (approx. 80 dB) during fear conditioning testing so they are not deaf. The degeneration of the circuitry controlling the acoustic startle reflex may be another potential causal factor for the ASR deficit observed. Indeed, markers of neurodegeneration and axonal injury are observed in the brainstem of 8.5-week-old *SOD1*^*G93 A*^ mice (Evans et al. 2014; Kim et al. 2011), a region known to be involved in eliciting ASR [reviewed in (Yeomans and Frankland 1995)]. An effect of CBD on ASR was observed in 19-week-old mice, with CBD specifically decreasing the ASR in *SOD1*^*G93 A*^ males further. Anxiolytic drugs [CBD is known to have some anxiolytic properties, at least in acute treatment designs (Guimarães et al. 1990; Long et al. 2012; Watt et al. 2020)] can decrease acoustic startle without impacting on PPI (Abduljawad et al. 1997). However, the effect of CBD in our study was sex- and genotype-specific, suggesting an overall reduction in anxiety may not be responsible for this effect, as we found the opposite effect in the OF at 12 weeks of age and no effect on OF anxiety at 18 weeks of age. In this context, it should be noted that bodyweight differences can affect ASR, however, without impacting on PPI (Fodor et al. 2016; Kraeuter et al. 2019).

PPI was lower in all *SOD1*^*G93 A*^ mice in line with previous studies (Guerra et al. 2021; Kreilaus et al. 2020), potentially demonstrating a behavioural consequence of serotonergic abnormalities that are evident in ALS patients and *SOD1*^*G93 A*^ transgenic mice (Turner et al. 2003, 2005). It should be noted that other signalling pathways are also known to impact on PPI (e.g. dopaminergic and glutamatergic circuits) and, thus, could be involved in the observed PPI deficit (Bakshi and Geyer 1998; Zhang et al. 2000). Importantly, CBD is a known 5-HT_1 A_ agonist (Campos and Guimarães 2008; Russo et al. 2005) and is able to partially restore amphetamine-induced PPI deficits in mice (Pedrazzi et al. 2015). This mechanism may play a role in restoring the PPI response we observed in CBD-treated female *SOD1*^*G93 A*^ mice, however, it should be noted the effect observed was moderate—an interaction driven by the 86 dB prepulse in young female transgenics, which was not evident in older females.

Testing fear-associated memory, all mice responded similarly during the conditioning trial. Interestingly, female *SOD1*^*G93 A*^ exhibited higher levels of total *freezing* during the context and cue trials regardless of CBD treatment. The sex-specific effect we observed here may be derived from the effects of sex hormones on this behavioural domain. Specifically, estrogen has been found to increase fear-associated *freezing* in mice (Morgan and Pfaff 2001), and activation of estrogen receptor alpha (ERα) and beta (ERβ) disrupts inhibition of fear in rats (Toufexis et al. 2007). Interestingly, ERα and ERβ are upregulated in reactive astrocytes and microglia in the spinal cord of symptomatic *SOD1*^*G93 A*^ mice (McLeod et al. 2020). It is possible that these changes also occur in the brains of *SOD1*^*G93 A*^ mice and could partly explain the fear conditioning phenotype observed (i.e. combined effect of sex and *SOD1*^*G93 A*^ genotype in females). The importance of estrogen in *SOD1*^*G93 A*^ pathogenesis was also demonstrated in a study where ovariectomised female transgenic mice had reduced survival compared to sham (this was rescued by 17β-estradiol treatment) (Choi et al. 2008). Another potential mechanism involved in fear-associated *freezing* of *SOD1*^*G93 A*^ females could be an imbalanced serotonergic system. Mice lacking the 5-HT_1 A_ receptor have an increased *freezing* response to a conditioned context (Klemenhagen et al. 2006) and indeed, there is evidence of serotonergic dysfunction in *SOD1*^*G93 A*^ transgenic mice as well as in ALS patients (Dentel et al. 2013; Forrest et al. 1996; Sofic et al. 1991; Turner et al. 2003, 2005).

In the current study, CBD affected male mice only, increasing the *freezing* response in the cue trial. It is currently unclear why this effect was sex-specific. The influence of CBD may be exerted via 5-HT_1 A_ receptor binding as serotonergic signalling pathways in the amygdala are important in fear conditioning (reviewed in (Bocchio et al. 2016)). However, the exact influence of serotonin signalling on fear is not completely clear as acute and chronic studies have discovered opposite effects when using selective serotonin reuptake inhibitor treatment (Burghardt et al. 2004; Grillon et al. 2007). Our chronic study provides additional evidence that a 5-HT_1 A_ agonist (CBD) may increase the *freezing* response in male mice.

CBD and THC have distinct receptor profiles, with THC primarily binding to cannabinoid receptors 1 (CB_1_) and 2 (CB_2_) whereas CBD exhibits very low displacement activities at those cannabinoid receptors and can function as an inverse agonist (although it may have indirect activation properties for CB_1_; reviewed in (Karl et al. 2017)). CBD also has other targets including G protein-coupled receptor 55 (GPR55), GPR18, and Transient Receptor Potential Vanilloid 1 (TRPV1) as well as 5-HT_1a_ receptors. Looking at the limited evidence from other studies on cannabinoid effects on *SOD1*^*G93 A*^ transgenic mice, THC and the synthetic CB_1_/CB_2_ agonist WIN55,212–2 (WIN) delayed disease progression although WIN-treatment did not affect the life span of ALS transgenic mice (Bilsland et al. 2006; Raman et al. 2004). When investigating the role of CB_2_ separately, conflicting insights have been gained with the CB_2_ agonist AM-1241 providing either short-term delay in motor symptom progression without affecting bodyweight or survival (Kim et al. 2006) or increasing the survival interval after disease onset by over 50% (Shoemaker et al. 2007). More research needs to be carried out in the field to more comprehensively assess the behavioural effects of cannabinoid treatment on disease symptoms of ALS mouse models and to clarify the role of the various receptor systems.

One limitation of the current study is the lack of food intake data for the experimental groups. However, individual housing would have been required for an accurate measurement of food consumption and thereby interfered with the behavioural assessment study focus. Importantly, previous reports on food intake in *SOD1*^*G93 A*^ mice are inconsistent and the recording method is often inaccurate (Cocozza et al. 2021; Dupuis et al. 2004; Steyn et al. 2020). Steyn and coworkers used an automated home cage system; food intake was similar pre-symptomatically (see also (Dupuis et al. 2004)) and only increased in individually housed ALS transgenic mice from week 16 onwards. Thus, the CBD intake across sex and genotype of the current study was similar for most of the experimental period and the weekly motor performance data do not suggest increased CBD effects in the last 4 weeks of testing. Finally, our approach is in line with other studies using dietary treatments, which were not adjusted for changing bodyweights or potential differences in food intake (Dupuis et al. 2004; Zhao et al. 2012).

In conclusion, the study discovered beneficial effects of oral CBD on the bodyweight deficit in both male and female *SOD1*^*G93 A*^ mice as well as improving social recognition memory and the PPI response in female *SOD1*^*G93 A*^ mice. CBD also reduced the ASR and increased the *freezing* response to a conditioned cue in both *SOD1*^*G93 A*^ transgenic and WT male mice. However, CBD treatment did not reverse motor impairments or sensorimotor gating deficits. Thus, chronic oral CBD treatment at the dose administered here may be therapeutically useful for only particular ALS symptoms including bodyweight decline, which is an indicator of disease progression and declining survival rate (Dharmadasa et al. 2017). Further investigations should consider additional CBD dosing and beginning treatment at an earlier age prior to the onset of motor deficits. This could be followed by combination treatments of CBD and e.g. cannabinoid receptor antagonists to explore potential mechanisms behind observed CBD effects.

## Supplementary Information

Below is the link to the electronic supplementary material.

Supplementary file1 (DOCX 767 KB)

Supplementary file2 (DOCX 16 KB)

## Data Availability

Data will be made available on request.
