# Supplementary material for: Behavioural effects of oral cannabidiol (CBD) treatment in the superoxide dismutase 1 G93 A (SOD1G93 A) mouse model of amyotrophic lateral sclerosis
Source: Psychopharmacology (Berl). 2025 Apr 14;242(9):2077–95. doi: 10.1007/s00213-025-06785-z (PMC12380930; doi:10.1007/s00213-025-06785-z)
Supplement: Supplementary file 1 — Supplementary file1 (DOCX 767 KB) [file 213_2025_6785_MOESM1_ESM.docx]

Statistical analysis for pole test (Supplementary Figure 1)

No genotype effects were detected in either sex in the first week of testing (11 weeks of age) for either parameter investigated (i.e. latency to reach platform, latency to reach platform once turned). In male mice, three-way RM ANOVA revealed an ‘age’ by ‘genotype’ interaction [F(8,248) = 7.48, *p* < 0.0001] as *SOD1^G93A^* transgenic mice regardless of treatment took increasingly longer to reach the bottom compared to WT males (no ‘age’ by ‘genotype’ by ‘treatment’ interaction, *p* > 0.05; **Fig. S1A**). A similar ‘age’ by ‘genotype’ interaction [F(8,248) = 2.72, *p* = 0.007] was evident when analysing the latency to reach the platform once turned (no ‘age’ by ‘genotype’ by ‘treatment’ interaction, *p* > 0.05; **Fig. S1C**). A strong trend for an ‘age’ by ‘treatment’ interaction [F(8,248) = 1.97, *p* = 0.05] indicated that pole test performance across weeks was better in CBD-fed males compared to controls (**Fig. S1C**).

In females, no significant main effects or interactions were detected when analysing the latency to reach the platform (all *p’s* > 0.05; **Fig. S1B**). However, an ‘age’ by ‘genotype’ interaction for the latency to reach the platform once turned [F(8,288) = 2.11, *p* = 0.035] indicated that *SOD1^G93A^* transgenic females also developed a motor impairment across weeks when compared to WT mice (no ‘age’ by ‘genotype’ by ‘treatment’ interaction, *p* > 0.05; **Fig. S1D**). CBD had no effects on the pole test performance of females (all *p’s* > 0.05).

Supplementary Figures

**Supplementary Figure 1A**


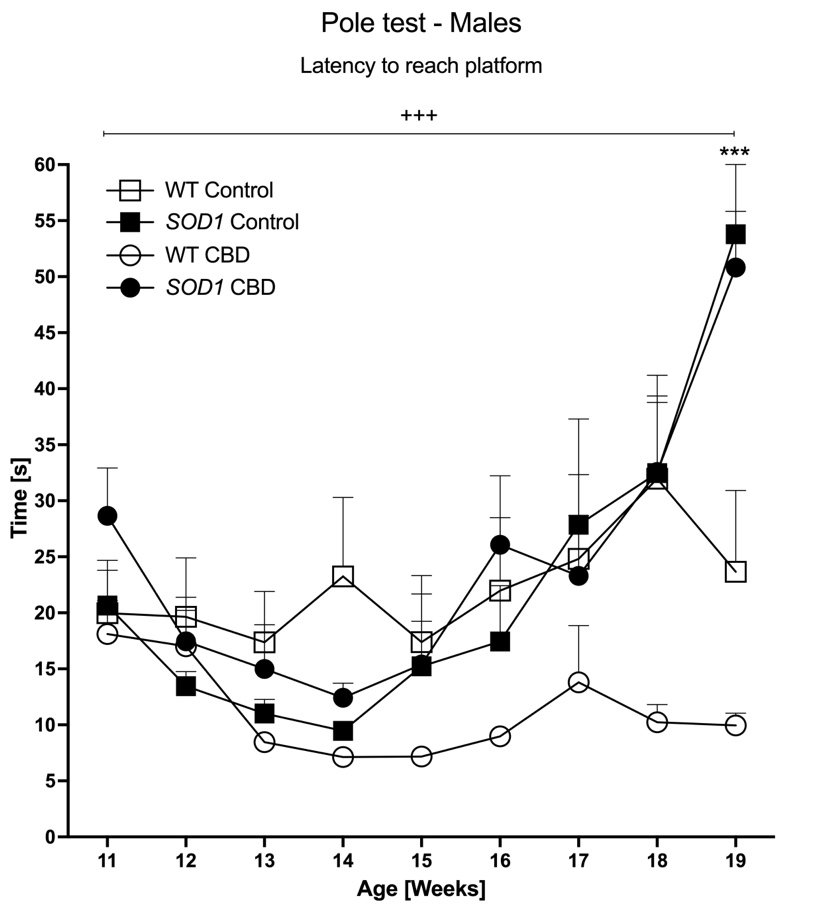


**Supplementary Figure 1B**

**
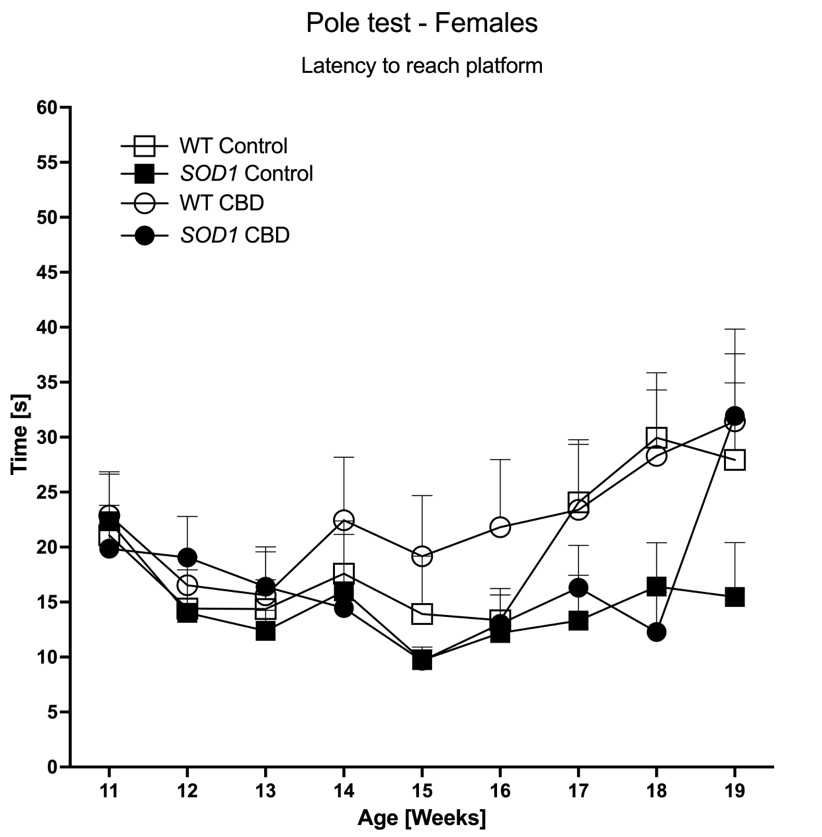
**

**Supplementary Figure 1C**

**
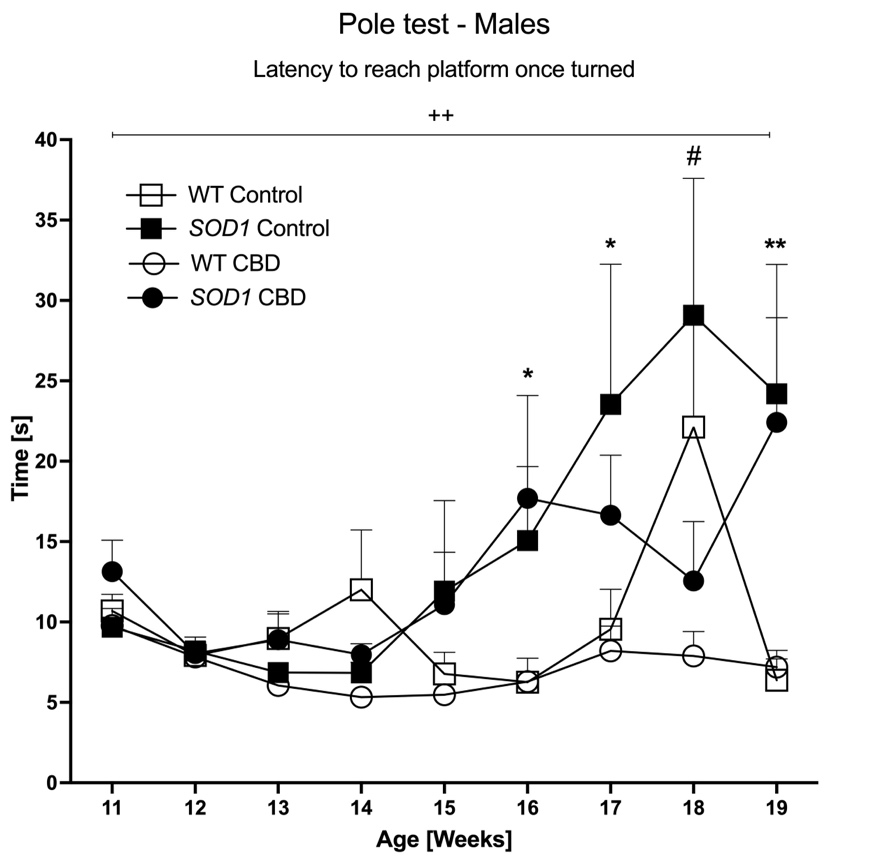
**

**Supplementary Figure 1D**


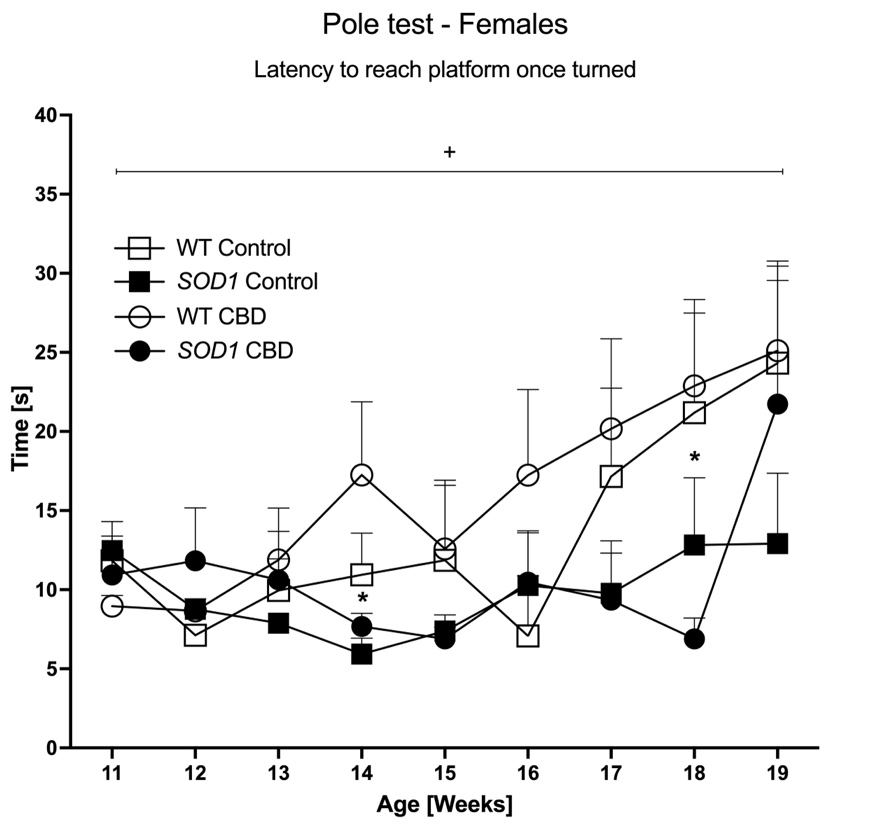


###### Supplementary Figure 1A-D - Motor function in the pole test: Latency [s] to (A-B) reach the platform or (C-D) reach the platform once turned are shown. (A/C) Male and (B/D) female *SOD1^G93A^* transgenic (*SOD1^G93A^*) and wild type-like (WT) mice were chronically fed with either a CBD-enriched (CBD) or standard chow (Control) diet. Data are shown as mean + SEM. Three-way RM ANOVA ‘age’ by ‘genotype’ interactions are indicated by ^+^*p* < 0.05, ^++^*p* < 0.01, ^+++^*p* < 0.0001. For each week, two-way ANOVA main effects of ‘genotype’ across treatment groups are indicated by ^*^*p* < 0.05, ^**^*p* < 0.01, ^***^*p* < 0.0001 and ‘treatment’ effects across genotype are indicated by ^#^*p* < 0.05.

**Supplementary Figure 2A**


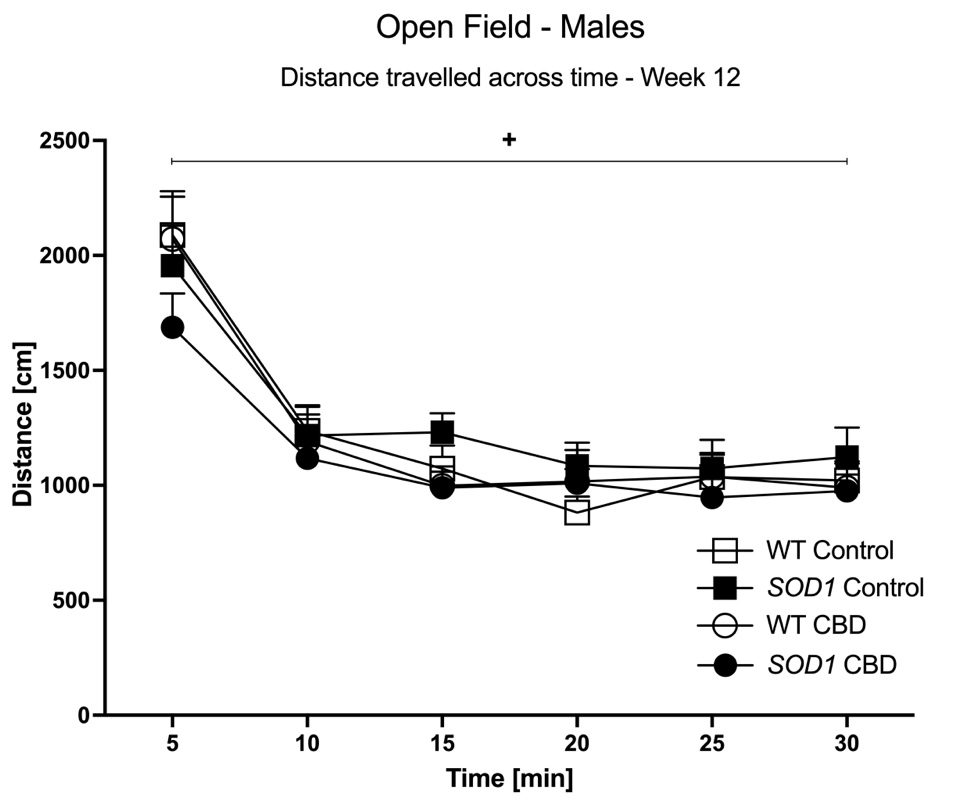


**Supplementary Figure 2B**

**
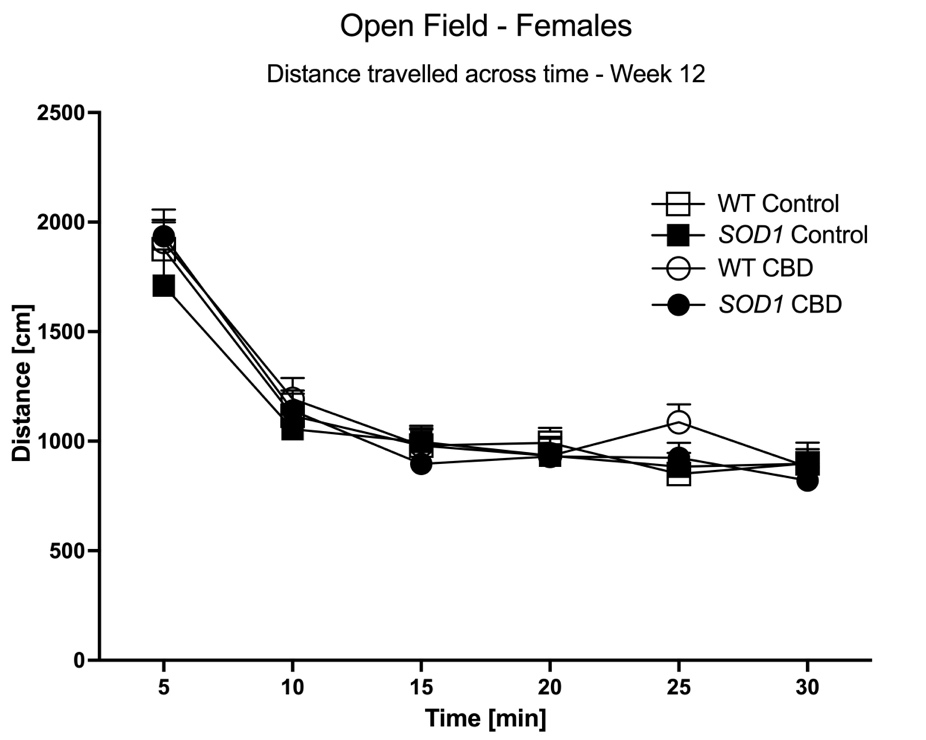
**

**Supplementary Figure 2C**


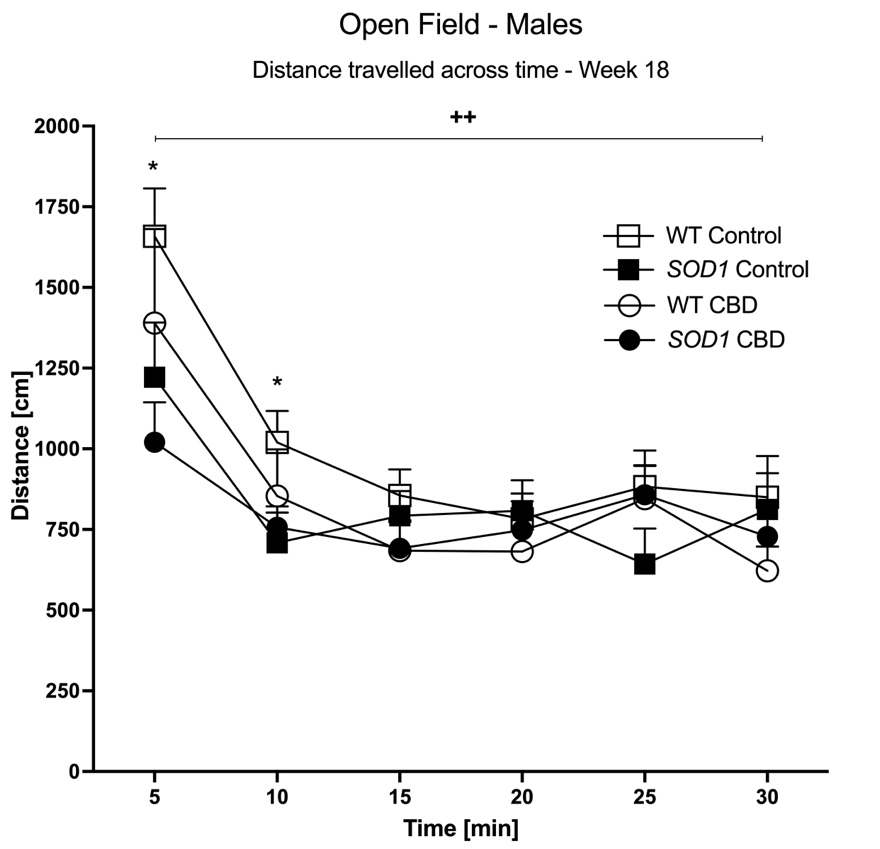


**Supplementary Figure 2D**


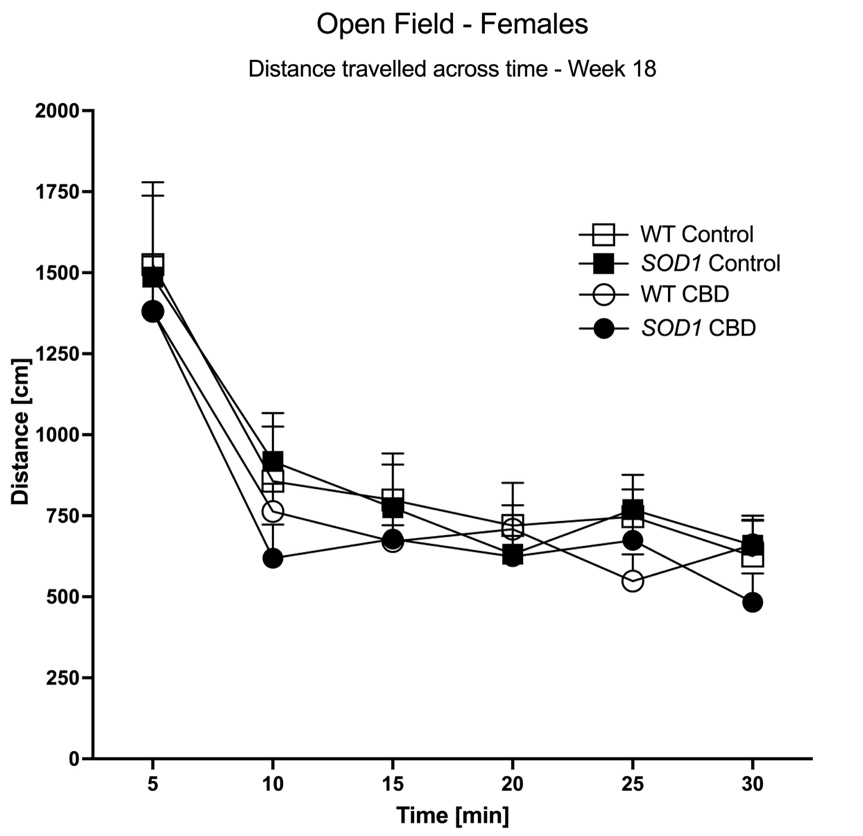


###### Supplementary Figure 2A-D - Open field locomotion: Distance travelled [cm] across 5-min blocks at (A/B) 12 weeks and (C/D) 18 weeks of age is shown. (A-C) Male and (B-D) female *SOD1^G93A^* transgenic (*SOD1^G93A^*) mice and wild type-like (WT) mice were chronically fed with either a CBD-enriched (CBD) or standard chow (Control) diet. Data are shown as mean + SEM. Three-way RM ANOVA ‘time’ by ‘genotype’ interactions in males are indicated by ^+^*p* < 0.05 and ^++^*p* < 0.01. Two-way ANOVA ‘genotype’ effects across treatment groups for 5-min blocks are indicated by **p* < 0.05.
