# Supplementary material for: Behavioural effects of oral cannabidiol (CBD) treatment in the superoxide dismutase 1 G93 A (SOD1G93 A) mouse model of amyotrophic lateral sclerosis
Source: Psychopharmacology (Berl). 2025 Apr 14;242(9):2077–95. doi: 10.1007/s00213-025-06785-z (PMC12380930; doi:10.1007/s00213-025-06785-z)
Supplement: Supplementary file 2 — Supplementary file2 (DOCX 16 KB) [file 213_2025_6785_MOESM2_ESM.docx]

| **Test parameter and effect or interaction** | ***p*-values** |
| --- | --- |
| Bodyweight across weeks: Main sex effect | <0.0001 |
| Bodyweight across weeks: Sex*age*treatment interaction | 0.024 |
| Accelerod –latency to fall: Sex*genotype interaction | 0.044 |
| Pole test – latency to reach platform: Sex*genotype interaction | 0.013 |
| Pole test – latency to reach platform: Sex*age*genotype interaction | <0.0001 |
| Pole test – latency to reach platform once turned: Sex*age*genotype interaction | 0.001 |
| Open field- centre zone locomotion ratio (12 weeks): Sex*genotype*treatment interaction | 0.002 |
| Open field- centre zone time (12 weeks): Sex*genotype*treatment interaction | 0.04 |
| Open field – 30 min locomotion (18 weeks): Sex*time*genotype interaction | 0.01 |
| Acoustic startle response (13 weeks): Sex*startle pulse*genotype interaction | 0.001 |
| Acoustic startle response (19 weeks): Sex*startle pulse*genotype interaction | <0.0001 |
| Prepulse inhibition (13 weeks): Sex*prepulse*genotype interaction | 0.025 |
| Fear conditioning – context test: Sex*genotype interaction | 0.04 |
| Fear conditioning – cue test: Sex*time interaction | <0.0001 |

**Supplementary Table 1 – Sex effects:** Main effects of ‘sex’ and interactions with ‘sex’ identified during analysis of the behavioural test data. *P*-values derived from either two or three-way ANOVAs, or two, three or four-way RM ANOVA (in case of interactions with ‘age’, ‘time’, ‘startle pulse’, and ‘prepulse’).

|  | **WT Control** | ***SOD1* Control** | **WT CBD** | ***SOD1* CBD** |
| --- | --- | --- | --- | --- |
| **Males** – Week 18  Centre time [s] | 181.94 ± 36.42 | 205.91 ± 41.21 | 180.99 ± 81.17 | 110.64 ± 24.60 |
| **Females** – Week 18  Centre time [s] | 133.81 ± 35.07 | 104.39 ± 19.49 | 69.79 ± 15.99 | 71.98 ± 19.05 |
| **Males** – Week 18  Percentage centre locomotion [%] | 25.78 ± 3.12 | 24.25 ± 2.60 | 21.97 ± 6.43 | 15.00 ± 2.31 |
| **Females** – Week 18  Percentage centre locomotion [%] | 21.46 ± 3.81 | 16.14 ± 3.08 | 14.75 ± 2.27 | 13.40 ± 2.97 |

**Supplementary Table 2 – Anxiety-like behaviours in the open field at 18 weeks of age:** Time spent in centre [s] as well as percentage centre locomotion [%] in *SOD1^G93A^* (*SOD1*) transgenic and wild type-like (WT) littermates, treated with either a CBD-enriched (CBD) or standard chow (Control) diet. Data presented as means ± SEM.
